# Mapping the Human Leukocyte Antigen Diversity among Croatian Regions: Implication in Transplantation

**DOI:** 10.1155/2021/6670960

**Published:** 2021-04-07

**Authors:** Zorana Grubic, Marija Maskalan, Katarina Stingl Jankovic, Marija Burek Kamenaric, Renata Zunec

**Affiliations:** Tissue Typing Centre, Clinical Department for Transfusion Medicine and Transplantation Biology, University Hospital Centre Zagreb, Zagreb, Croatia

## Abstract

In the present study, HLA allele and haplotype frequencies were studied using the HLA data of 9277 Croatian unrelated individuals, typed using high-resolution methods for the HLA-A, -B, -C, and -DRB1 loci. The total numbers of observed alleles were 47 for HLA-A, 88 for HLA-B, 34 for HLA-C, and 53 for HLA-DRB1. HLA-A^∗^02:01 (29.5%), B^∗^51:01 (10.5%), C^∗^04:01 (15.8%), and DRB1^∗^16:01 (10.4%) were the most frequent alleles in the Croatian general population. The three most frequent haplotypes were HLA-A^∗^01:01~C^∗^07:01~B^∗^08:01~DRB1^∗^03:01 (4.7%), HLA-A^∗^03:01~C^∗^07:02~B^∗^07:02~DRB1^∗^15:01 (1.7%), and HLA-A^∗^02:01~C^∗^07:01~B^∗^18:01~DRB1^∗^11:04 (1.5%). Allele and haplotype frequencies were compared between national and regional data, and differences were observed, particularly in the North Croatia region. The data has potential use in refining donor recruitment strategies for national registries of volunteer hematopoietic stem cell donors, solid organ allocation schemes, and the design of future disease and anthropological studies.

## 1. Introduction

The Human Leukocyte Antigen (HLA) genes have been the focus of numerous studies in the past decades to their key role in processes of immune recognition; on the one hand, and an extensive polymorphism reflected in both the large number of genes and their immense allelic variety on the other. A very large proportion of these studies have been population studies, since knowledge about the HLA polymorphism in a given population has extensive applications, among which solid organ transplantation and allogeneic hematopoietic stem cell transplantation (allo-HSCT) from an unrelated donor are one of the most important ones.

The importance of HLA polymorphism in solid organ transplantation arises from the direct correlation between the transplantation outcome and HLA matching of the recipient and the donor. Since the probability of a matched donor grows with the higher level of the HLA diversity among the deceased donor pool, the need for establishing international organisations for allocation and cross-border exchange of deceased donor organs, such as Eurotransplant, became evident early on.

The development of the allo-HSCT program is highly dependent on the existence of volunteer HSC donor registries since these registries provide an HLA-matched unrelated donor (MUD) for those patients who do not have an HLA-identical sibling. Moreover, the number of HSCTs preformed from an unrelated donor is constantly increasing worldwide, as well as in Croatia, and it is closely followed by the parallel improvements and the expansion of volunteer HSC donor registries. Currently, there are more than 37 million volunteer HSC donors and cord blood units around the world, recruited in almost 80 national registries and more than 50 cord blood banks which list their donors/CBUs in the World Marrow Donor Association (WMDA) database [1]. The Croatian Bone Marrow Donors Registry (CBMDR) was founded in 1993 and joined the Bone Marrow Donors Worldwide (BMDW) organisation in the same year. As of August 2020, CMBDR enlists almost 60000 unrelated HSC donors.

The national HSC donor registry databases with HLA profiles of enrolled donors are a valuable source of information regarding the HLA polymorphism of a population to which these donors belong and can be used to enhance the strategies for registry improvement and development. Namely, one of the critical questions in the policy of each registry is that of size or more precisely of the sufficient/optimal number of donors needed for an efficient national registry. The second question is how to obtain this number, which leads to the necessity of developing optimised recruitment plans for increasing the number of donors [2–5]. Aside from increasing the number of donors in general, numerous strategies can be employed depending on the end-goal. Enrolment of younger male individuals is an example of such a strategy. This policy has been adopted by various registries, prompted by studies which have shown a correlation between donor age and survival after HSCT, e.g., patients who received HSC from younger donors had a better survival rate [6]. The advantages of male versus female donors have not been unequivocally proven by similar studies thus far, although in theory, female donors are less preferable due to possible HLA sensitisation as a result of pregnancy.

On the other hand, an effective recruitment and selection strategy based on HLA allele and haplotype frequencies can be established. Moreover, differences in HLA profiles of donors recruited in different donor recruitment centres may be representative of regional diversity [2, 3]. Population studies as well as registry data demonstrated that a detailed characterization of the HLA polymorphisms in different populations worldwide is important and required in the field of allo-HSCT [7–9]. Several previous studies focused on HLA diversity at the regional level in national registries demonstrated that HLA frequencies vary across different geographic regions and are correlated with geography [9, 10]. Conversely, other similar studies have been published with reports of no such regional HLA differences observed [11]. These results suggest that recruitment strategies may differ from one country to another. Motivated by these opposing data, the question was raised of whether regional differences in HLA polymorphism exist in Croatia. Croatia's placement on the Balkan Peninsula as well as the observed influence of various other populations on the Croatian population in certain parts of the country would suggest that such differences could be expected. Croatians migrated from the Baltic to South East Europe in the 7th century. In that early period, a part of the Croatian population settled on the Adriatic coast which was followed by mixing with the South East Europe's autochthons (Illyrians, Thracians). Afterward, in the last few centuries, northern and central parts of Croatia were influenced by Austrians, Hungarians, and Germans while the southern part was influenced by Italians, as well as by Turks [12].

**Figure 1:**
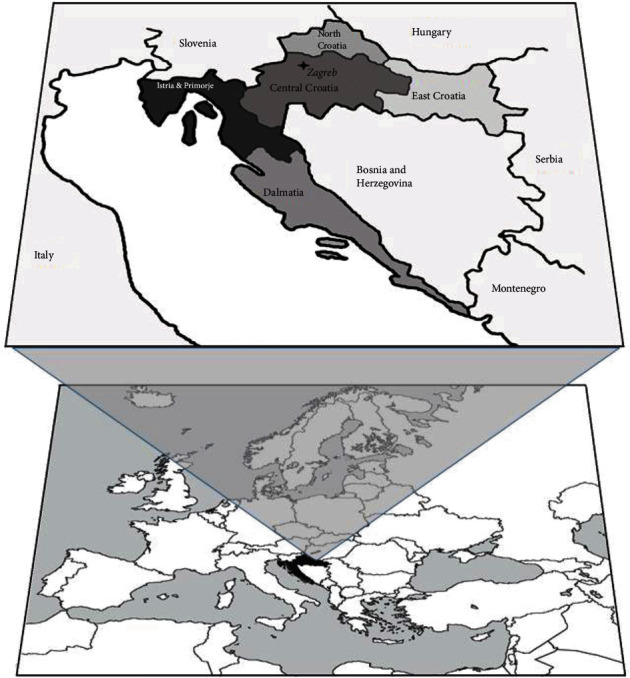
Map of Europe and Croatia. Legend: Central Croatia region varies greatly in population density, as it includes areas with the highest population density (Zagreb, Croatia's capital and surroundings) but also the mountainous areas of Gorski Kotar, Lika, which represent the least populated area in Croatia; East Croatia—the lowland area of the Pannonian Plain, bordered by Croatia's largest rivers, Sava, Drava, and Danube; North Croatia—a small, moderately populated, hilly region on the northernmost border of Croatia; Istria & Primorje includes Istria, the narrow Kvarner region, and nearby islands; Dalmatia—Mediterranean region, with three distinguishable parallel belts: the islands, the coast, and the hinterland.

Regarding Croatia's regions, the contemporary regional division of the country into the northern and southern parts is essentially dictated by the country's geographical features. The northern, predominantly lowland part of the county, is then divided into Central Croatia, East Croatia, and North Croatia, while the coastal, southern part of Croatia is usually subdivided into two regions: Istria & Primorje and Dalmatia (Figure 1). Along with the geographic feature, this division also takes into account the influence of different populations on these regions throughout history [12].

In order to expand the current knowledge about the HLA polymorphism in the Croatian population and explore the possibility of regional differences in HLA allele and haplotype distribution, data from CBMDR were used in the present study. Although information about HLA allele and haplotype frequencies among Croatians has already been reported in a few publications, those analyses have focused only on the Croatian population as whole, without taking into account the different regions of Croatia [13, 14].

## 2. Material and Methods

### 2.1. Subjects

A total of 9277 volunteer unrelated donors from the CBMDR were included in the dataset (data extracted on 01-12-2018). The donors were recruited and originate from 5 different regions of Croatia (1532 from Dalmatia, 865 from Istria & Primorje, 1021 from Central Croatia, 1877 from East Croatia, and 1049 from North Croatia) as well as from Zagreb (*N* = 2933), as illustrated by Figure 1. The number of samples chosen for each region correlates with its population size and represents 0.2% of the number of inhabitants for a specific region. The number of towns included for each region is as follows: 11 from Dalmatia, 8 from Istria & Primorje, 10 from Central Croatia, 9 from East Croatia, and 8 from North Croatia. Donors from the same region were then selected based on their residence in different towns situated in a given region, in such a way that all towns were adequately represented. Finally, individuals residing in the same town and carrying the same surname were excluded from the sample. The regions do not represent official Croatian counties, but rather a geographical division of Croatia. One possible disadvantage of the sample selection method used in this study is the potential gene flow due to population migrations, which regularly occur in Croatia in the direction of a regional centre of each region. For that reason, the capital city of Croatia, Zagreb, was excluded from the sample of the Central Croatia region since constant migration to Zagreb occurs from all Croatian regions. For the same reason, the sample including subjects residing in Zagreb was chosen as a sample representative of the Croatian population in general. The study was approved by the Ethics Committee of the University Hospital Zagreb, and it was performed in line with the Helsinki Declaration.

### 2.2. HLA Typing

Genomic DNA was isolated from 5 mL of peripheral blood, with EDTA collected from each donor, using a commercial kit (MagNA Pure LC DNA; Roche Diagnostics GmbH, Mannheim, Germany).

The data analyzed in the present study consisted of the results obtained by molecular typing of CBMDR donors at HLA-A, -C, -B, and -DRB1 loci using the PCR-SSO (Polymerase Chain Reaction Sequence Specific Oligo probes) method (Lifecodes HLA SSO, Gen Probe Transplant Diagnostics Inc, Stamford, CT, USA, or LABType SSO, One Lambda, MA, USA). This method uses kits for HLA-A, -B, and -C which cover exons 2 and 3, while kits for HLA-DRB1 cover exon 2. The 5′ ends of upstream primers (included in kits) were labelled with biotin, and each PCR product was hybridized with a different number of probes for each HLA locus, complementary to the polymorphic sequences. After hybridization, amplicons were labelled with streptavidin-R-phycoerythrin, which is a specific fluorescent ligand of biotin, and quantified on the Luminex LABScanTM 100 flow analyzer (Luminex Corporation, Austin, TX, USA). Results were analyzed using a corresponding software for analysis (MatchIT DNA software v. 1.2 or HLA Fusion Software v. 4.3) [15]. The high-resolution typing results were obtained using the Polymerase Chain Reaction Sequence Specific Primers (PCR-SSP) method (CareDx, Stockholm, Sweden), and results were processed using the Helmberg SCORE 5 software [16]. HLA ambiguous typing results were retested by employing the sequence-based typing (PCR-SBT) method using a commercial Olerup SBT Resolver kit (CareDx Pty Ltd, Fremantle Western Australia, Australia) [17]. Data obtained by the SBT method were evaluated by Olerup Assign SBT v. 4.7.1 program. The IPD-IMGT/HLA databases 3.31.0-3.35.0 were used for analysis.

In cases when HLA ambiguous results were still present, a decision was made based on the CWD allele data [18]. The list of HLA ambiguities that could not be resolved using the abovementioned methods is available in Supplementary Table 1. It is necessary to mention that by using this approach, we perhaps failed to detect some rare or very rare alleles, but the projected percentage of such cases is very low.

### 2.3. Statistical Analysis

Allele frequencies were calculated using the GeneRate program [19] and also by direct counting, with no difference observed in results obtained by these two approaches. In cases when only one allele was present at a given locus, the individual was counted as homozygous. PyPop (PyThon for Population genetics, version 0.7.0) was used to test for Hardy–Weinberg equilibrium (HWE), to conduct the Ewens-Watterson homozygosity analysis and to estimate four-locus haplotype frequencies [20]. The significance of differences in allele and haplotype frequencies between regions was evaluated using the chi-square test, while Fisher's exact test with Yates correction was used if any of the values in 2 × 2 tables were <5 (GraphPad QuickCalcs online software, https://www.graphpad.com/). *P* value was corrected by the number of alleles observed at each locus (*P*corr). *P* values obtained for haplotype analysis were also corrected for multiple testing.

## 3. Results

The expected and observed allele frequencies for the alleles at tested HLA loci did not differ significantly, and populations from all regions as well as the population from Zagreb were found in the Hardy–Weinberg equilibrium (Table 1). As the first part of the analysis, we compared the alleles observed among individuals included in the present study with the most recent catalogue of common and well-defined (CWD) alleles by the European Federation for Immunogenetics (EFI) [18]. This comparison revealed that 165 (81.7%) of HLA alleles detected in this study have been included in the EFI CWD catalogue. Conversely, 37 (18.3%) alleles found in the Croatian population have not been reported as CWD alleles in the EFI CWD catalogue thus far.

In the second part of the study, the analysis of the HLA allele frequency distribution was performed. Ten most frequent alleles at tested HLA loci in Zagreb and their respective frequencies in each region are listed in Table 2.

A comparison of the data obtained for the sample from Zagreb with the results from the previously published study for the Croatian population did not reveal any statistically significant difference, which justified our choice of Zagreb data as reference data for our population in general [13].

A total of 47 HLA-A alleles were found in our entire sample (*N* = 9277), among which the most frequent allele in the Zagreb population was A^∗^02:01 (29.5%). This allele was also the most frequent allele in all five analyzed regions, with a frequency ranging from 28.2% to 31.5%. The second-ranked allele by frequency in Zagreb was HLA-A^∗^01:01 (12.9%), which appeared among individuals from five regions with a frequency ranging from 11.7% to 14.0%. Finally, with a frequency of 11.8%, which placed it in the third place in the Zagreb sample, the allele HLA-A^∗^03:01 was detected in the five regions with a frequency ranging from 10.1% to 11.6%. The HLA-B locus exhibited the highest polymorphism with 88 detected alleles, of which the three most frequent alleles among Zagreb residents were HLA-B^∗^51:01 (10.5%; frequency range in the five regions from 8.3% to 12.6%), HLA-B^∗^18:01 (8.0%; ranging from 7.5% to 9.5% in the regions), and HLA-B^∗^07:02 (7.5%; frequency ranges from 6.6% to 8.0% in the five tested regions). Among 34 different alleles observed at HLA-C locus, two alleles, HLA-C^∗^04:01 (ranging from 13.5% to 16.7%) and C^∗^07:01 (ranging from 14.1% to 17.0%), were present in more than 10.0% of the tested subjects in each region, while the third HLA-C allele ranked by frequency in the Zagreb sample, HLA-C^∗^12:03 (11.6%), exhibited a frequency range from 8.5% to 14.9% among subjects from the five regions. Fifty-three different alleles were determined at the HLA-DRB1 locus. Three most frequent alleles among Zagreb citizens were HLA-DRB1^∗^16:01 (10.4%; ranging from 7.2% to 13.0% in the five regions), HLA-DRB1^∗^03:01 (10.1%; ranging from 9.9% to 11.7% in the five regions), and HLA-DRB1^∗^01:01 (10.0%; ranging from 8.1% to 10.7% in the five regions). The distribution of all observed alleles at four tested HLA loci in the Zagreb population as well as in each region is listed in Supplementary Table 2.

**Figure 2:**
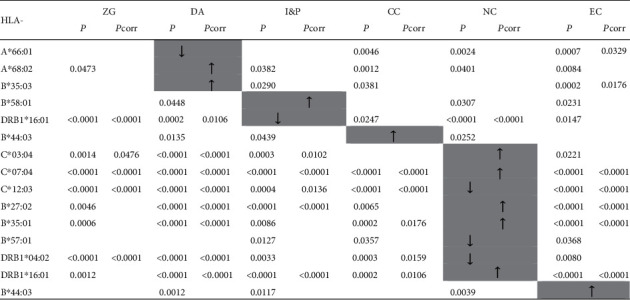
The list of HLA-A, -C, -B, and -DRB1 alleles which demonstrated a significantly different frequency in one region in comparison to at least three other regions. Legend: ZG: Zagreb; DA: Dalmatia; I&P: Istria & Primorje; CC: Central Croatia; NC: North Croatia; EC: East Croatia; *P*corr: corrected *P* value by the number of alleles observed at each locus; ↑/↓: frequency significantly higher/lower than in at least three other regions; grey-shaded box: region differing in allele frequency from at least three other regions.

Figure 2 summarizes the data about 17 alleles whose frequencies significantly deviated (were either increased or decreased) in one region in comparison to at least three other regions (Zagreb is excluded). The highest number of these alleles (*N* = 10), for four of which (HLA-C^∗^07:04, C^∗^12:03, DRB1^∗^04:02, and DRB1^∗^16:01) the observed differences remain significant even after the correction of *P* value, was detected in the North Croatia region. It is interesting to note that the occurrence of two alleles belonging to the same gene group (HLA-DRB1^∗^04:01 and DRB1^∗^04:02) was significantly different in this region in comparison to the other parts of Croatia. This investigation also revealed that the difference in the distribution between regions can be attributed to only two HLA-A alleles (HLA-A^∗^66:01 and A^∗^68:02). These alleles were present with a significantly different frequency in Dalmatia in comparison to other regions. The results of the analysis also revealed that different alleles of the same gene group exhibit significant variation in frequency among subjects from five Croatian regions. Examples of such variation are the HLA-B^∗^35 alleles: the HLA-B^∗^35:01 allele was significantly more frequent in North Croatia, while the HLA-B^∗^35:03 allele was more frequent in Dalmatia. The HLA-B^∗^35:08 allele was observed with a significantly higher frequency in Istria & Primorje but only in comparison to Dalmatia and North Croatia, and therefore, it was not included in Figure 2. In contrast, the HLA-B^∗^35:02 allele was present with similar frequencies in all regions (from East Croatia - 1.1% to North Croatia - 1.7%). Another example is the frequency of the HLA-B^∗^44:03 allele, which was significantly higher in Central Croatia and East Croatia than in the rest of Croatia.

The fourth aim of the present study was to analyze the distribution of non-CWD alleles (according to data presented in the EFI CWD catalogue—version 1.0) and to evaluate their distribution in different Croatian regions [18]. As suggested in a previous study and to avoid possible misinterpretations, we used an additional term, “local” (LOC), to categorize the alleles which occurred ≥3 times in our sample but are not present in the current EFI CWD catalogue [21].

**Figure 3:**
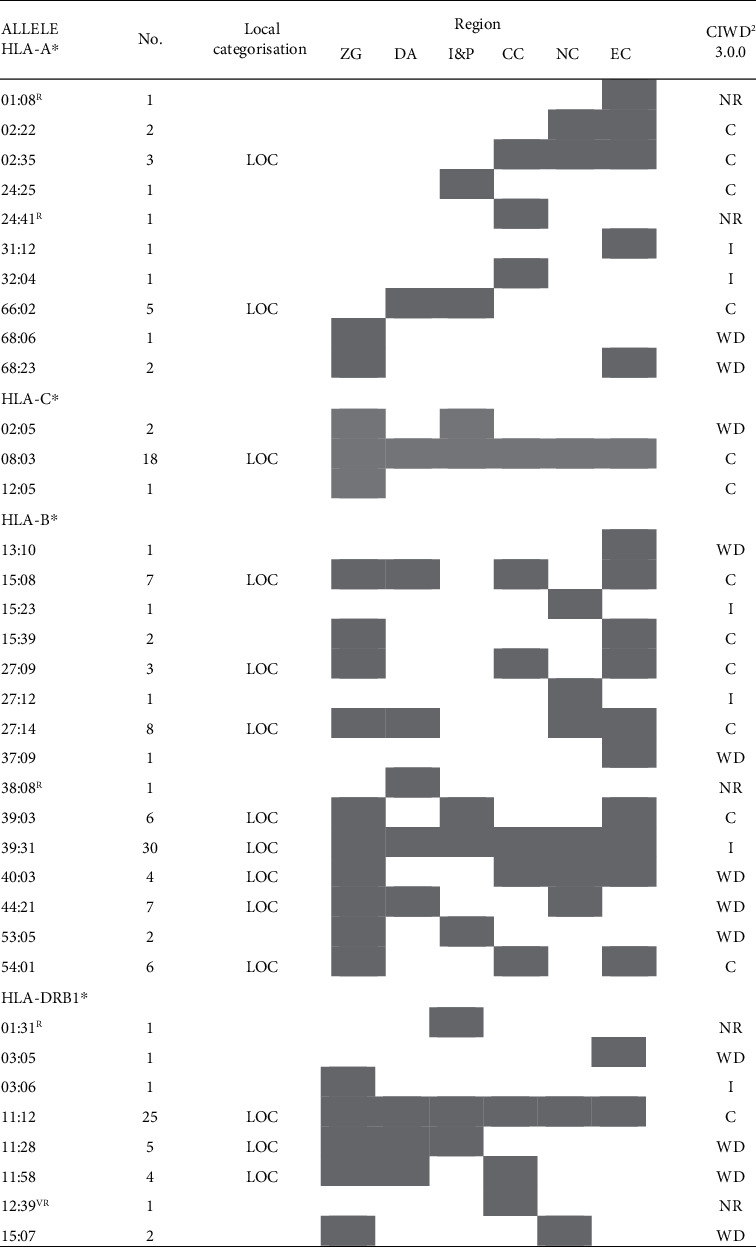
The distribution of non-CWD^1^ alleles in different regions of Croatia. Legend: ZG: Zagreb; DA: Dalmatia; I&P: Istria & Primorje; CC: Central Croatia; EC: East Croatia; NC: North Croatia; No: number of occurrence in total sample; ^1^common and well-documented alleles according to the EFI CWD catalogue [18]; ^R^rare allele according to rare allele detector (RAD) [21]; ^VR^very rare allele according to RAD^21^; grey/white box: allele is present/absent in the region; LOC: alleles occurred ≥3 times in our sample but are not present in the EFI CWD catalogue [18]; grey-shaded box: allele observed; ^2^reference [23]; C: common; I: intermediate; NR: not reported; WD: well-documented.

Figure 3 lists LOC alleles, but also all other HLA alleles observed ≤2 times in this study. Among 15 HLA alleles observed only once, one third (5 out of 15) were found in East Croatia, three alleles were detected in Central Croatia, two in North Croatia, one in Dalmatia, and Istria & Primorje each, and the remaining two alleles with one occurrence appeared among the residents of Zagreb. In this group of HLA alleles observed only once in our study, four are classified as rare (HLA-A^∗^01:08, A^∗^24:41, B^∗^38:08, and DRB1^∗^01:31), while DRB1^∗^12:39 is categorized as very rare according to the Rare Alleles Detector (RAD) [22]. It is interesting to mention that among twelve non-CWD alleles classified as LOC alleles, only three alleles (HLA-B^∗^39:31, C^∗^08:03, and DRB1^∗^11:12) were observed in all regions. Finally, the comparison of our LOC alleles with the new CIWD 3.0.0 catalogue revealed that no data exists in this catalogue for five alleles (HLA-A^∗^01:08, A^∗^24:41, B^∗^38:08, DRB1^∗^01:31, and DRB1^∗^12:39). On the other hand, 14 alleles classified as LOC in our sample, which are not listed in the EFI CWD catalogue as CWD alleles, appear as well-documented alleles (HLA-B^∗^44:21, DRB1^∗^11:28, and DRB1^∗^11:58), intermediate alleles (HLA-B^∗^39:31 and B^∗^40:03), or common ones (HLA-A^∗^02:35, A^∗^66:02, B^∗^15:08, B^∗^27:09, B^∗^27:14, B^∗^39:03, B^∗^54:01, C^∗^08:03, and DRB1^∗^11:12) in the CIWD 3.0.0 catalogue [18, 23].

The three most frequent HLA-A~C~B~DRB1 haplotypes with a frequency >1.0% in all regions were HLA-A^∗^01:01~C^∗^07:01~B^∗^08:01~DRB1^∗^03:01 (range from 4.3%, Central Croatia to 6.4%, Dalmatia), HLA-A^∗^03:01~C^∗^07:02~B^∗^07:02~DRB1^∗^15:01 (range from 1.3%, Dalmatia to 1.8%, Central Croatia), and HLA-A^∗^02:01~C^∗^07:01~B^∗^18:01~DRB1^∗^11:04 (range from 1.4%, North Croatia to 2.1%, Central Croatia). The distribution of the remaining 20 most frequent HLA-A~C~B~DRB1 haplotypes in Zagreb, which represents the Croatian population in total, is presented for each region in Table 3. The remaining HLA-A~C~B~DRB1 haplotypes found ≥4 times in each region are shown in Supplementary Table 3.

**Figure 4:**
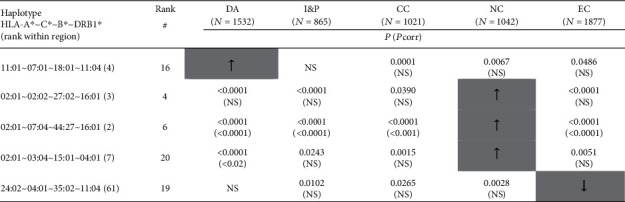
The list of HLA-A~C~B~DRB1 haplotypes among 20 most frequent in the general Croatian population, which demonstrated a significantly different frequency in one region in comparison to the at least three other regions. Legend: ^#^Zagreb/general Croatian population; DA: Dalmatia; I&P: Istria & Primorje; CC: Central Croatia; EC: East Croatia; NC: North Croatia; ↑/↓: frequency significantly higher/lower than in at least three other regions; grey-shaded box: region differing in haplotype frequency from at least three other regions; *P*: value for comparison of specific region differing in haplotype frequency from at least three other regions; *P*corr: *P* value corrected for multiple testing; NS: not significant

For five out of the 20 most frequent HLA-A~C~B~DRB1 haplotypes among Croatians in general, a significantly different frequency was observed in one region in comparison to at least three other regions (Figure 4). Again, the highest number of such haplotypes was found in the North Croatia region.

## 4. Discussion

The present study is the first analysis of the HLA polymorphism in different regions of Croatia. The regions included in this analysis correspond to the established regional division of Croatia, and the number of samples pertaining to each region was adjusted according to the population size for that particular region. The comparison of HLA allele and haplotype distribution was performed between different regions as well as between each region and the Croatian population in general (as represented by the Zagreb sample).

As no deviation from the HWE was detected, our registry sample may be considered as representative for the regional population as suggested by different authors [24, 25].

Comparison of allele frequencies at tested HLA loci between the Zagreb sample and our previous study, which included 4000 individuals from different cities, has not revealed any significant difference and therefore additionally supports our hypothesis that Zagreb data can be used as reference data for the Croatian population in total [13]. At the same time, some differences were observed between five Croatian regions as well as between regions and the Zagreb data.

The observed HLA heterogeneity of the Croatian population is probably a result of a very turbulent history during which numerous influences on different populations in different regions of Croatia occurred. For example, prior to the arrival of the Slavic population as a part of the Avar migration, the area of Dalmatia was inhabited by different Illyric tribes which mixed with Greek colonists, especially on the islands [26]. This substratum was later on Romanised as part of the Roman Empire. As part of the coastal area in the vicinity of Italy, Istria was constantly exposed to the influences and admixing which arrived from the Apennine area, even after the fall of the Roman Empire [12]. In the northern parts of Croatia, the Illyric population mixed with the Celts before the Romans, and after the fall of the Roman Empire, traces of the migration period (of different Germanic tribes) remained in this area. This area of Croatia was a part of different political associations in the later periods, and as a result, traces of the former Austro-Hungarian Monarchy population are visible (Germans, Hungarians, Austrians, and Czechs). The central part of Croatia as well as the easternmost parts shows traces of the long-term Ottoman presence. An additional possible cause for the genetic heterogeneity of the Croatians is the country's location in South-Eastern Europe on the corridor between Southern and Northern Europe. Genetic drift probably also added to the variation among the different regions. Finally, the variation could likewise be a result of a selection caused by the exposure to different pathogens and a subsequent better or worse adaptation of individuals with specific HLA genes.

Ten most frequently observed alleles represent approximately 90% of the cumulative frequency at the HLA-A locus; this percentage was nearly 65% at the HLA-B locus, around 85% for the HLA-C locus, while at the HLA-DRB1 locus, their frequency amounted to 78%. The HLA-A^∗^02:01 allele was the most frequent allele at the HLA-A locus in all regions, and the comparison of this allele between our and neighbouring populations did not reveal any significant difference [22]. Only six out of 88 different HLA-B alleles showed a frequency >5.0%, and the HLA-B^∗^51:01 allele was the most frequent one in almost all regions (except North Croatia). This study supports the data from a previous investigation which showed that HLA-B^∗^51:01 is the most frequent among HLA-B alleles. The same finding was reported for some other populations in the south of Europe [22]. Regarding the HLA-C locus, the HLA-C^∗^07:01 allele was more frequent in Dalmatia, Istria & Primorje, and East Croatia compared to Central and North Croatia, whereas the HLA-C^∗^04:01 allele was more common in Central and North Croatia in comparison to the rest of the country. In general, the frequency of the HLA-C^∗^07:01 allele increases from Southern (Spain - 11.0%) to Northern Europe (United Kingdom - 19.0%), and the frequencies detected for this allele among individuals from Croatian regions fall somewhere in the middle of these values (around 15%). The opposite observation was made for the HLA-C^∗^04:01 allele, whose frequency decreases in the same south-north direction, and, once again, the Croatian regional frequencies from 13.5% to 16.7% fit well in that range [22].

One of the interesting results pertaining to the HLA-DRB1 locus is the low frequency of the HLA-DRB1^∗^16:01 allele (7.2%) in Istria & Primorje, which is perhaps caused by the marked influence of the Italian population on this region. Namely, the frequency of the HLA-DRB1^∗^16:01 allele among Italians is around 5.0% [22]. This allele occurs among individuals from the remaining regions of Croatia with a frequency of around 10.0% or higher (North Croatia - 13.0%). This frequency distribution fits well with results from other population studies which state that the DRB1^∗^16:01 allele can be found with the highest frequency in Southern European countries (e.g., 13.7% among Greeks, 14.9% among North Macedonians, or as much as 15.5% among Bulgarians) [22]. North Croatia is also a very peculiar region regarding the distribution of the two most frequent HLA-DRB1^∗^04 alleles (HLA-DRB1^∗^04:01 and DRB1^∗^04:02).

HLA allele frequency distribution demonstrated that differences between the regions are not large; however, it nevertheless disclosed a number of HLA alleles with significant differences among the regions. Seven alleles were present in North Croatia with a significant difference in frequency when compared to all other regions as well as in comparison to the Croatian population in general. This finding is probably associated with the geographic specificity of that region and its relative isolation from the other regions. Namely, even in a small country such as Croatia, there was some migration of population in the past, for example, from Dalmatia to East Croatia. For the North Croatia region, however, there were never reports of substantial migration in either direction.

According to the EFI CWD catalogue ver. 1, the percentage of non-CWD HLA alleles in the Croatian population was 17.9%. Among these non-CWD alleles, a few alleles (HLA-B^∗^39:31, C^∗^08:03, and DRB1^∗^11:12) are present in all Croatian regions and fall into the LOC allele category for our population, despite the fact that they are not even classified as WD in the abovementioned catalogue. The data about non-CWD alleles in our population raises the assumption that we probably failed to detect some rare and very rare HLA alleles, but published data for other populations so far suggest that this percentage is undoubtedly very low.

This discrepancy might exist due to the criteria for establishing common status in the EFI CWD catalogue and further corroborates the suggestion that populations where these alleles are commonly observed are underrepresented in the EFI CWD catalogue since no data for them were available at the time the catalogue was established. This is especially the case for the populations from the South-Eastern region of Europe. The matter of population size could also be involved in the explanation of the fact that some LOC alleles in Croatia are not even listed as WD alleles in EFI CWD catalogue [18]. Namely, there is a considerable difference in the sample size of the Croatian population represented in the EFI CWD catalogue and the one reported in the present study. The sample of the current study is at least twice as large, and it is expected that less frequent alleles will have a better chance of being detected in a larger population study. Our study points out some of the differences in the lists of available CWD catalogues published so far [18, 23]. Also, it highlights that different population data pools provide different information about the categorization of HLA alleles in the group of CWD alleles [18, 23]. After all, this is the reason why the inclusion of more population data for HLA alleles is important for obtaining a precise image about CWD alleles and for gaining more information on HLA diversity.

The data presented in this study show that all regions share the same three most frequent haplotypes, while the differences begin with the fourth-ranked haplotypes. The haplotype HLA-A^∗^01:01~C^∗^07:01~B^∗^08:01~DRB1^∗^03:01 is the most frequent haplotype in the Croatian population in total as well as in all five regions; this is also the case in majority of European populations [22]. The analysis of the origin of 20 most frequent HLA haplotypes, as suggested by Martinez-Laso et al., revealed that the majority (*N* = 15) fit into the category of Western European or Euro Mediterranean haplotypes. Out of the remaining five haplotypes, three belong to common Mediterranean haplotypes (HLA-A^∗^33:01~C^∗^08:02~B^∗^14:02~DRB1^∗^01:02, HLA-A^∗^01:01~C^∗^12:02~B^∗^52:01~DRB1^∗^15:02, and HLA-A^∗^24:02~C^∗^04:01~B^∗^35:02~DRB1^∗^11:04), one haplotype is classified as Northern-European (HLA-A^∗^02:01~C^∗^07:01~B^∗^08:01~DRB1^∗^03:01), and one is originated from Phoenician-Berbers (HLA-A^∗^33:01~C^∗^08:02~B^∗^14:02~DRB1^∗^03:01) [27].

Nonetheless, as described in the results, some HLA four-locus haplotypes exhibit specific regional characteristics. For example, three haplotypes were significantly more frequent in North Croatia in comparison to all other regions. Haplotype HLA-A^∗^02:01~C^∗^03:04~B^∗^15:01~DRB1^∗^04:01, ranked 20^th^ in the total population, was located in the 7^th^ place, haplotype HLA-A^∗^02:01~C^∗^07:04~B^∗^44:27~DRB1^∗^16:01 was second, while haplotype HLA-A^∗^02:01~C^∗^02:02~B^∗^27:02~DRB1^∗^16:01 was third. The data about HLA polymorphisms obtained in this study is valuable for resolving the HLA diversity in different regions of Croatia, and it could be a valid tool for developing a new recruitment strategy for CBMDR. Moreover, in solid organ transplantation setting, our recent study emphasized the importance of including populations with different HLA profiles in international organ exchange programs [28]. More precisely, that study suggested that, for example, patients on a waiting list for kidney transplantations who are HLA-B^∗^18 positive had a greater chance of receiving a kidney graft from a Croatian deceased donor then from the Eurotransplant donor pool. Finally, the presented data has a potential for use in designing HLA disease and anthropology studies as well.

To conclude, regardless of the fact that the Croatian population, in the global context, represents a very small population, HLA diversity can still be observed and therefore should be considered and documented.

## Figures and Tables

**Table 1:** Polymorphism at HLA-A, -C, -B, and -DRB1 loci in the Croatian population (*N* = 9277): heterozygosity parameters and categorization according to the EFI CWD catalogue^18^.

Region	Locus HLA-	Number of alleles				Heterozygosity			
		Total	COM^#^	WD^#^	Non-CWD	Obs	Exp	*P*	*P* value (overall)^1^
Zagreb (*N* = 2933)	A	33	27	4	2	2498	2512.44	0.7733	0.1976
	C	30	24	3	3	2694	2661.49	0.5285	0.1723
	B	67	51	8	8	2803	2783.14	0.7066	0.8336
	DRB1	47	38	4	5	2716	2729.20	0.8005	0.0820

Dalmatia (*N* = 1532)	A	31	26	4	1	1315	1321.02	0.8684	0.1563
	C	28	24	3	1	1411	1385.08	0.4861	0.4134
	B	58	47	6	5	1443	1444.37	0.9713	0.6171
	DRB1	41	35	3	3	1424	1423.30	0.9851	0.0449

Istria & Primorje (*N* = 865)	A	31	27	2	2	755	750.29	0.8633	0.5863
	C	28	24	2	2	784	782.15	0.9473	0.1225
	B	54	48	3	3	816	818.74	0.9238	0.6098
	DRB1	39	35	1	3	809	804.72	0.8801	0.7262

Central Croatia (*N* = 1021)	A	29	25	1	3	875	877.56	0.9312	0.1421
	C	28	25	2	1	936	928.49	0.8052	0.1935
	B	63	51	7	5	980	970.34	0.7565	0.7258
	DRB1	39	35	1	3	932	950.82	0.5416	0.0238

North Croatia (*N* = 1049)	A	30	26	2	2	884	890.47	0.8284	0.9194
	C	26	23	2	1	963	952.85	0.7424	0.0628
	B	53	44	3	6	998	994.55	0.9129	0.9486
	DRB1	41	36	3	2	971	969.97	0.9736	0.9290

East Croatia (*N* = 1877)	A	38	29	4	5	1623	1621.34	0.9672	0.0873
	C	27	24	2	1	1683	1700.18	0.6769	0.2344
	B	70	51	7	10	1780	1782.94	0.9445	0.1501
	DRB1	41	36	3	2	1741	1746.54	0.8946	0.0305

Legend: *N*: number of tested individuals; *n*: number of observed HLA alleles; Het Obs: observed heterozygosity; Het Exp: expected heterozygosity; ^**#**^COM: common alleles according to the EFI catalogue; ^**#**^WD: well-documented alleles according to the EFI catalogue^18^; ^1^overall *P* value for the Guo and Thompson HW test.

**Table 2:** The frequency of the 10 most frequent HLA-A, -C, -B, and -DRB1 alleles in Zagreb and their respective frequencies in five different Croatian regions.

	ZG (*N* = 2933)	DA (*N* = 1532)	I&P (*N* = 865)	CC (*N* = 1021)	NC (*N* = 1049)	EC (*N* = 1877)
	AF	AF	AF	AF	AF	AF
HLA-A^∗^						
02:01	0.2946	0.2830	0.2688	0.2933	0.3146	0.2818
01:01	0.1289	0.1407	0.1400	0.1166	0.1168	0.1396
03:01	0.1176	0.1005	0.1081	0.1156	0.1096	0.1018
24:02	0.1149	0.1106	0.1243	0.1185	0.1149	0.1119
11:01	0.0660	0.0839	0.0723	0.0622	0.0758	0.0765
26:01	0.0496	0.0460	0.0468	0.0593	0.0419	0.0519
32:01	0.0436	0.0473	0.0393	0.0397	0.0429	0.0418
68:01	0.0411	0.0437	0.0486	0.0387	0.0367	0.0397
25:01	0.0292	0.0274	0.0278	0.0289	0.0253	0.0312
23:01	0.0252	0.0186	0.0214	0.0294	0.0167	0.0237
∑	0.9107	0.9017	0.8974	0.9022	0.8952	0.8999

HLA-C^∗^						
04:01	0.1572	0.1354	0.1457	0.1455	0.1668	0.1428
07:01	0.1488	0.1697	0.1688	0.1410	0.1506	0.1630
12:03	0.1161	0.1377	0.1202	0.1219	0.0848	0.1244
02:02	0.0931	0.0875	0.0786	0.1024	0.0958	0.0916
06:02	0.0835	0.0809	0.0983	0.0931	0.0720	0.0924
07:02	0.0803	0.0702	0.0809	0.0769	0.0877	0.0717
01:02	0.0452	0.0597	0.0486	0.0480	0.0405	0.0461
03:03	0.0416	0.0333	0.0382	0.0407	0.0453	0.0418
05:01	0.0413	0.0415	0.0347	0.0382	0.0400	0.0424
15:02	0.0373	0.0415	0.0457	0.0358	0.0329	0.0330
∑	0.8444	0.8574	0.8597	0.8435	0.8164	0.8492

HLA-B^∗^						
51:01	0.1045	0.1257	0.1133	0.1033	0.0834	0.0951
18:01	0.0796	0.0950	0.0850	0.0901	0.0748	0.0908
07:02	0.0752	0.0672	0.0711	0.0705	0.0801	0.0655
08:01	0.0736	0.0917	0.0925	0.0651	0.0791	0.0842
35:01	0.0643	0.0450	0.0642	0.0568	0.0872	0.0570
35:03	0.0549	0.0601	0.0451	0.0465	0.0367	0.0496
38:01	0.0438	0.0542	0.0468	0.0529	0.0324	0.0450
15:01	0.0416	0.0304	0.0382	0.0407	0.0543	0.0421
27:05	0.0397	0.0385	0.0364	0.0426	0.0343	0.0440
13:02	0.0346	0.0304	0.0347	0.0313	0.0329	0.0360
∑	0.6118	0.6382	0.6273	0.5998	0.5952	0.6093

HLA-DRB1^∗^						
16:01	0.1037	0.1031	0.0723	0.0931	0.1301	0.0923
03:01	0.1006	0.1168	0.1127	0.0989	0.1063	0.1052
01:01	0.0999	0.0809	0.0948	0.0955	0.1072	0.0980
15:01	0.0902	0.0943	0.0942	0.0823	0.0739	0.0866
07:01	0.0885	0.0774	0.0890	0.0989	0.0820	0.0996
11:01	0.0796	0.0708	0.0809	0.0916	0.0834	0.0821
11:04	0.0767	0.0842	0.0942	0.0788	0.0777	0.0773
13:01	0.0597	0.0754	0.0647	0.0544	0.0577	0.0594
13:02	0.0418	0.0539	0.0457	0.0426	0.0405	0.0346
08:01	0.0292	0.0258	0.0225	0.0289	0.0300	0.0285
∑	0.7699	0.7826	0.7710	0.7650	0.7888	0.7636

Legend: ZG: Zagreb; DA: Dalmatia; I&P: Istria & Primorje; CC: Central Croatia; NC: North Croatia; EC: East Croatia; AF: allele frequency.

**Table 3:** The distribution of the twenty most frequent HLA-A~C~B~DRB1 haplotypes in Zagreb and five different regions of Croatia (three most frequent haplotypes are not included).

Haplotype HLA-A^∗^~C^∗^~B^∗^~DRB1^∗^	Haplotype frequency (%)					
	Zagreb (*N* = 2933)	Dalmatia (*N* = 1532)	Istra & Primorje (*N* = 865)	Central Croatia (*N* = 1021)	North Croatia (*N* = 1049)	Slavonia (*N* = 1877)
02:01~02:02~27:02~16:01	11.7	7.1	5.8	14.3	22.8	9.4
11:01~04:01~35:01~01:01	11.1	8.6	12.0	6.1	8.0	5.6
02:01~07:04~44:27~16:01	10.6	6.4	0.6	5.4	25.3	7.1
03:01~04:01~35:01~01:01	10.6	5.5	11.5	10.1	14.2	6.8
02:01~06:02~13:02~07:01	9.8	9.1	8.8	14.6	9.5	10.7
23:01~04:01~44:03~07:01	9.4	6.5	8.1	10.4	7.4	10.5
02:01~02:02~27:05~01:01	8.6	8.5	7.3	5.0	8.7	9.9
26:01~12:03~38:01~04:02	7.4	8.4	8.0	10.2	0	5.7
25:01~12:03~18:01~15:01	7.3	8.2	6.9	6.4	5.7	8.6
33:01~08:02~14:02~01:02	6.5	4.2	5.1	5.9	8.1	5.6
02:01~07:02~07:02~15:01	6.0	4.5	6.5	9.9	8.0	5.4
02:01~06:02~57:01~07:01	5.5	7.1	7.9	4.5	0	5.0
02:01~07:01~08:01~03:01	4.7	4.6	8.7	1.0	6.3	3.3
01:01~12:02~52:01~15:02	4.5	3.3	5.2	4.4	3.8	4.2
33:01~08:02~14:02~03:01	4.5	2.9	2.4	3.43	6.8	2.1
02:01~05:01~44:02~04:01	4.3	5.5	4.1	2.5	3.0	4.0
24:02~04:01~35:02~11:04	3.8	3.6	6.3	5.4	7.1	1.9

*N*: number of subjects.

## Data Availability

The data used to support the findings of this study are available from the corresponding author upon request

## Supplementary Materials

Supplementary 1 sTable 1: the list of ambiguities.

Supplementary 2 sTable 2: the distribution of HLA alleles in Zagreb area and five Croatian regions.

Supplementary 3 sTable 3: the distribution of HLA haplotypes in Zagreb area and five Croatian regions.
