# Supplementary material for: Mapping the Human Leukocyte Antigen Diversity among Croatian Regions: Implication in Transplantation
Source: J Immunol Res. 2021 Apr 7;2021:6670960. doi: 10.1155/2021/6670960 (PMC8051524; doi:10.1155/2021/6670960)
Supplement: Supplementary 1 — sTable 1: the list of ambiguities. [file 6670960.f1.doc]

**s Table 1. The list of ambiguites**
