# Supplementary material for: Mapping the Human Leukocyte Antigen Diversity among Croatian Regions: Implication in Transplantation
Source: J Immunol Res. 2021 Apr 7;2021:6670960. doi: 10.1155/2021/6670960 (PMC8051524; doi:10.1155/2021/6670960)
Supplement: Supplementary 2 — sTable 2: the distribution of HLA alleles in Zagreb area and five Croatian regions. [file 6670960.f2.doc]

**s Table 2. The distribution of HLA-A, -C, -B, -DRB1 alleles in Zagreb area and five Croatian regions**

|  |  |  |  |  |  |  |
| --- | --- | --- | --- | --- | --- | --- |
| **REGION** | **Zagreb**  **(N=2933)**  **AF** | **Dalmatia**  **(N=1532)**  **AF** | **Istria & Primorje**  **(N=865)**  **AF** | **Central Croatia**  **(N=1021)**  **AF** | **North Croatia**  **(N=1049)**  **AF** | **East Croatia**  **(N=1877)**  **AF** |
| **HLA-A*** |  |  |  |  |  |  |
| **01:01** | 0.12888 | 0.14067 | 0.13988 | 0.11655 | 0.11678 | 0.13958 |
| **01:02** | 0.00017 | - | 0.00058 | - | 0.00048 | - |
| **01:03** | - | - | 0.00116 | - | - | 0.00053 |
| **01:08** | - | - | - | - | - | 0.00027 |
| **01:200** | - | 0.00033 | - | - | - | - |
| **02:01** | 0.29458 | 0.28296 | 0.26879 | 0.29334 | 0.31459 | 0.28183 |
| **02:02** | 0.00119 | 0.00098 | 0.00231 | 0.00196 | - | 0.00133 |
| **02:03** | - | - | - | - | - | 0.00027 |
| **02:05** | 0.00528 | 0.00816 | 0.01040 | 0.00930 | 0.01049 | 0.00986 |
| **02:06** | 0.00136 | 0.00163 | 0.00116 | - | 0.00286 | 0.00213 |
| **02:11** | - | 0.00033 | - | - | 0.00048 |  |
| **02:17** | 0.00068 | 0.00033 | - | - | - | 0.00107 |
| **02:22** | - | - | - | - | 0.0004 | 0.00027 |
| **02:35** | - | - | - | 0.00049 | 0.0004 | 0.00027 |
| **03:01** | 0.11763 | 0.10052 | 0.10809 | 0.11557 | 0.10963 | 0.10176 |
| **03:02** | 0.00426 | 0.00816 | 0.00867 | 0.00490 | 0.00238 | 0.00533 |
| **11:01** | 0.06597 | 0.08388 | 0.07225 | 0.06219 | 0.07579 | 0.07645 |
| **23:01** | 0.02523 | 0.01860 | 0.02139 | 0.02938 | 0.01668 | 0.02371 |
| **23:02** | - | - | - | - | - | 0.00027 |
| **24:02** | 0.11473 | 0.11064 | 0.12428 | 0.11851 | 0.11487 | 0.11188 |
| **24:03** | 0.00222 | 0.00490 | 0.00636 | 0.00196 | 0.00048 | 0.00346 |
| **24:25** | - | - | 0.00058 | - | - | - |
| **24:41** | - | - | - | 0.00049 | - | - |
| **25:01** | 0.02915 | 0.02742 | 0.02775 | 0.02889 | 0.02526 | 0.03117 |
| **26:01** | 0.04961 | 0.04602 | 0.04682 | 0.05926 | 0.04194 | 0.05194 |
| **26:08** | 0.00034 | - | - | - |  | 0.00107 |
| **29:01** | 0.00273 | 0.00196 | 0.00173 | 0.00343 | 0.00191 | 0.00213 |
| **29:02** | 0.00597 | 0.00294 | 0.00405 | 0.00686 | 0.00286 | 0.00666 |
| **30:01** | 0.00938 | 0.00914 | 0.01156 | 0.01077 | 0.01573 | 0.00986 |
| **30:02** | 0.00188 | 0.00490 | 0.00347 | 0.00098 | 0.00334 | 0.00133 |
| **30:04** | 0.00239 | 0.00587 | 0.00462 | 0.00294 | 0.00143 | 0.00373 |
| **31:01** | 0.02114 | 0.02415 | 0.02197 | 0.02106 | 0.02383 | 0.01838 |
| **31:08** | - | - | - | - | - | 0.00027 |
| **31:11** | - | - | - | - | - | 0.00027 |
| **32:01** | 0.04364 | 0.04732 | 0.03931 | 0.03967 | 0.04290 | 0.04182 |
| **32:04** | - | - | - | 0.00049 | - | - |
| **33:01** | 0.02114 | 0.01240 | 0.01445 | 0.02302 | 0.02622 | 0.01785 |
| **33:03** | 0.00102 | 0.00131 | 0.00116 | 0.00147 | 0.00095 | 0.00107 |
| **34:02** | 0.00017 | - | - | - | 0.00095 | - |
| **66:01** | 0.00273 | 0.00098 | 0.00347 | 0.00539 | 0.00667 | 0.00533 |
| **66:02** | - | 0.00131 | 0.00058 | - | - | - |
| **68:01** | 0.04108 | 0.04373 | 0.04855 | 0.03869 | 0.03670 | 0.03969 |
| **68:02** | 0.00392 | 0.00718 | 0.00231 | 0.00098 | 0.00191 | 0.00453 |
| **68:06** | 0.00017 | - | - | - | - | - |
| **68:23** | 0.00051 | - | - | - | - | 0.00027 |
| **69:01** | 0.00034 | 0.00033 | 0.00058 | 0.00098 | 0.00095 | 0.00160 |
| **74:01** | 0.00034 | - | - | - | - | - |
| **80:01** | 0.00051 | 0.00981 | 0.00173 | 0.00049 | - | 0.00080 |
| **TOTAL** | 1 | 1 | 1 | 1 | 1 | 1 |
| **Legend:** | AF - allele frequency | |  |  |  |  |

| **REGION** | **Zagreb**  **(N=2933)**  **AF** | **Dalmatia**  **(N=1532)**  **AF** | **Istria & Primorje**  **(N=865)**  **AF** | **Central Croatia**  **(N=1021)**  **AF** | **North Croatia**  **(N=1049)**  **AF** | **East Croatia**  **(N=1877)**  **AF** |
| --- | --- | --- | --- | --- | --- | --- |
| **HLA-C*** |  |  |  |  |  |  |
| **01:02** | 0.04518 | 0.05973 | 0.04855 | 0.04799 | 0.04051 | 0.04608 |
| **02:02** | 0.09308 | 0.08747 | 0.07861 | 0.10235 | 0.09581 | 0.09164 |
| **02:05** | 0.00017 | - | 0.00058 | - | - | - |
| **03:02** | 0.00716 | 0.00587 | 0.01098 | 0.00881 | 0.00572 | 0.00479 |
| **03:03** | 0.04160 | 0.03329 | 0.03815 | 0.04065 | 0.04528 | 0.04182 |
| **03:04** | 0.02813 | 0.02023 | 0.02197 | 0.03183 | 0.04290 | 0.03117 |
| **04:01** | 0.15718 | 0.13544 | 0.14566 | 0.14104 | 0.16683 | 0.14278 |
| **05:01** | 0.04125 | 0.04145 | 0.03468 | 0.03820 | 0.04004 | 0.04235 |
| **05:08** | - | 0.00033 | - | - | 0.00048 | - |
| **06:02** | 0.08353 | 0.08094 | 0.09827 | 0.09305 | 0.07197 | 0.09243 |
| **07:01** | 0.14882 | 0.16971 | 0.16879 | 0.14545 | 0.15062 | 0.16303 |
| **07:02** | 0.08029 | 0.07017 | 0.08092 | 0.07689 | 0.08770 | 0.07166 |
| **07:04** | 0.02591 | 0.01632 | 0.01272 | 0.02106 | 0.04433 | 0.01811 |
| **07:07** | - | - | 0.00116 | - | 0.00048 | - |
| **07:17** | 0.00017 | - | - | - | - | - |
| **08:01** | 0.00051 | 0.00098 | 0.00058 | 0.00049 | 0.00095 | 0.00053 |
| **08:02** | 0.02711 | 0.02317 | 0.02428 | 0.02547 | 0.03051 | 0.02797 |
| **08:03** | 0.00102 | 0.00131 | - | 0.00098 | 0.00191 | 0.00053 |
| **12:02** | 0.01585 | 0.01338 | 0.01387 | 0.01273 | 0.01430 | 0.01572 |
| **12:03** | 0.11609 | 0.13773 | 0.12023 | 0.12194 | 0.08484 | 0.12440 |
| **12:05** | 0.00017 | - | - | - | - | - |
| **14:02** | 0.02608 | 0.03068 | 0.01965 | 0.01959 | 0.02097 | 0.01918 |
| **14:25** | - | - | - | - | - | 0.00027 |
| **15:02** | 0.03733 | 0.04145 | 0.04566 | 0.03575 | 0.03289 | 0.03303 |
| **15:04** | 0.00051 | 0.00033 | 0.00173 | 0.00049 | 0.00048 | 0.00053 |
| **15:05** | - | 0.00033 | 0.00116 | 0.00098 | - | 0.00053 |
| **15:06** | 0.00068 | 0.00131 | 0.00058 | 0.00098 | - | 0.00080 |
| **15:09** | 0.00017 | - | - | 0.00098 | - | - |
| **16:01** | 0.00580 | 0.00555 | 0.00058 | 0.01028 | 0.00286 | 0.00932 |
| **16:02** | 0.00614 | 0.00914 | 0.00694 | 0.00735 | 0.00429 | 0.00693 |
| **16:04** | 0.00239 | 0.00424 | 0.00751 | 0.00294 | 0.00381 | 0.00373 |
| **17:01** | 0.00324 | 0.00392 | 0.00289 | 0.00392 | 0.00286 | 0.00426 |
| **17:03** | 0.00409 | 0.00522 | 0.00520 | 0.00637 | 0.00667 | 0.00639 |
| **18:02** | 0.00034 | 0.00033 | 0.00173 | 0.00147 |  |  |
| **TOTAL** | 1 | 1 | 1 | 1 | 1 | 1 |
| **Legend:** | AF - allele frequency | |  |  |  |  |

| **REGION** | **Zagreb**  **(N=2933)**  **AF** | **Dalmatia**  **(N=1532)**  **AF** | **Istria & Primorje**  **(N=865)**  **AF** | **Central Croatia**  **(N=1021)**  **AF** | **North Croatia**  **(N=1049)**  **AF** | **East Croatia**  **(N=1877)**  **AF** |
| --- | --- | --- | --- | --- | --- | --- |
| **HLA-B*** |  |  |  |  |  |  |
| **07:02** | 0.07518 | 0.06723 | 0.07110 | 0.07052 | 0.08008 | 0.06553 |
| **07:02:03** | - | - | - | - | - | 0.00027 |
| **07:04** | 0.00051 | - | 0.00058 | 0.00098 | - | 0.00027 |
| **07:05** | 0.00222 | 0.00033 | 0.00289 | 0.00392 | 0.00286 | 0.00133 |
| **07:10** | - | - | - | 0.00098 | 0.00095 | - |
| **08:01** | 0.07364 | 0.09171 | 0.09249 | 0.06513 | 0.07912 | 0.08418 |
| **08:04** | - | - | - | - | - | 0.00027 |
| **13:02** | 0.03461 | 0.03035 | 0.03468 | 0.03134 | 0.03289 | 0.03596 |
| **13:10** | - | - | - | - | - | 0.00027 |
| **14:01** | 0.00494 | 0.00587 | 0.00462 | 0.00392 | 0.00572 | 0.00586 |
| **14:02** | 0.02267 | 0.01664 | 0.01965 | 0.02204 | 0.02526 | 0.02291 |
| **15:01** | 0.04160 | 0.03035 | 0.03815 | 0.04065 | 0.05434 | 0.04209 |
| **15:03** | 0.00085 | 0.00098 | 0.00058 | 0.00098 | - | 0.00053 |
| **15:07** | - | - | 0.00116 | - | - | 0.00053 |
| **15:08** | 0.00034 | 0.00033 | - | 0.00098 | - | 0.00053 |
| **15:09** | 0.00034 | - | - | 0.00049 | 0.00095 | 0.00053 |
| **15:10** | 0.00051 | 0.00098 | 0.00116 | 0.00147 | - | 0.00027 |
| **15:16** | 0.00017 | - | - | - | - | - |
| **15:17** | 0.00358 | 0.00457 | 0.00289 | 0.00392 | 0.00620 | 0.00320 |
| **15:18** | 0.00136 | 0.00098 | 0.00116 | 0.00294 | 0.00095 | 0.00107 |
| **15:23** | - | - | - | - | 0.00048 | - |
| **15:24** | 0.00068 | - | - | 0.00049 | 0.00048 | - |
| **15:39** | 0.00017 | - | - | - | - | 0.00027 |
| **18:01** | 0.07961 | 0.09497 | 0.08497 | 0.09011 | 0.07483 | 0.09084 |
| **18:03** | 0.00034 | 0.00033 | 0.00058 | 0.00049 | - | - |
| **18:05** | 0.00034 | - | - | 0.00049 | - | - |
| **18:37:02** | - | - | - | - | - | 0.00027 |
| **27:02** | 0.02523 | 0.01632 | 0.01329 | 0.02302 | 0.03765 | 0.01811 |
| **27:03** | - | - | - | 0.00049 | - | - |
| **27:05** | 0.03972 | 0.03851 | 0.03642 | 0.04261 | 0.03432 | 0.04395 |
| **27:07** | 0.00017 | 0.00033 | - | - | - | - |
| **27:09** | - | - | - | 0.00049 | - | 0.00053 |
| **27:12** | - | - | - | - | 0.00048 | - |
| **27:14** | 0.00051 | 0.00065 | - | - | 0.00048 | 0.00053 |
| **27:30** | - | - | - | - | - | 0.00080 |
| **35:01** | 0.06427 | 0.04504 | 0.06416 | 0.05681 | 0.08723 | 0.05701 |
| **35:02** | 0.01279 | 0.01208 | 0.01329 | 0.01273 | 0.01716 | 0.01066 |
| **35:03** | 0.05489 | 0.06005 | 0.04509 | 0.04652 | 0.03670 | 0.04955 |
| **35:04** | - | 0.00033 | - | - | - | 0.00053 |
| **35:08** | 0.00699 | 0.00849 | 0.01445 | 0.00930 | 0.01144 | 0.00773 |
| **37:01** | 0.00955 | 0.00555 | 0.01214 | 0.00833 | 0.01049 | 0.00879 |
| **37:09** | - | - | - | - | - | 0.00053 |
| **38:01** | 0.04381 | 0.05418 | 0.04682 | 0.05289 | 0.03241 | 0.04502 |
| **38:08** | - | 0.00033 | - | - | - | - |
| **39:01** | 0.02182 | 0.02970 | 0.03064 | 0.01616 | 0.01430 | 0.02504 |
| **39:03** | 0.00051 | - | 0.00058 | - | - | 0.00053 |
| **39:04** | 0.00136 | 0.00163 | 0.00058 | 0.00245 | 0.00048 | 0.00133 |
| **39:05** | - | - | 0.00058 | - | - | 0.00080 |
| **39:06** | 0.00392 | 0.00196 | 0.00289 | 0.00588 | 0.00429 | 0.00320 |
| **39:10** | 0.00017 | 0.00033 | - | 0.00098 | - | 0.00027 |
| **39:15** | - | - | - | 0.00049 | - | - |
| **39:24** | 0.00034 | 0.00033 | - | - | - | - |
| **39:31** | 0.00017 | - | 0.00116 | - | - | 0.00027 |
| **40:01** | 0.01534 | 0.01338 | 0.01098 | 0.01910 | 0.02336 | 0.01678 |
| **40:02** | 0.02097 | 0.02121 | 0.02023 | 0.02351 | 0.01716 | 0.02184 |
| **40:03** | - | - | - | 0.00049 | 0.00048 | 0.00053 |
| **40:06** | 0.00034 | 0.00098 | 0.00116 | 0.00098 | - | 0.00133 |
| **41:01** | 0.00409 | 0.00490 | 0.00289 | 0.00539 | 0.00429 | 0.00533 |
| **41:02** | 0.00443 | 0.00620 | 0.00578 | 0.00588 | 0.00667 | 0.00666 |
| **44:02** | 0,03750 | 0,03786 | 0,03237 | 0,03379 | 0,03622 | 0,03836 |
| **44:03** | 0.02608 | 0.02121 | 0.02197 | 0.03281 | 0.02145 | 0.03277 |
| **44:05** | 0.01193 | 0.00849 | 0.01156 | 0.00979 | 0.01096 | 0.01412 |
| **44:06** | - | - | - | - | - | 0.00053 |
| **44:21** | 0.00034 | 0.00098 | - | - | 0.00095 | - |
| **44:27** | 0,02489 | 0,01469 | 0,01330 | 0,02302 | 0,03957 | 0,01652 |
| **44:29** | - | 0.00033 | - | - | - | - |
| **45:01** | 0.00170 | 0.00196 | 0.00116 | 0.00196 | 0.00048 | 0.00213 |
| **46:01** | - | - | - | 0.00049 | - | - |
| **47:01** | 0.00119 | 0.00033 | 0.00058 | 0.00245 | 0.00143 | 0.00266 |
| **48:01** | 0.00153 | 0.00228 | 0.00058 | 0.00147 | 0.00286 | 0.00107 |
| **49:01** | 0.01943 | 0.01795 | 0.01503 | 0.01420 | 0.01525 | 0.01705 |
| **50:01** | 0.01125 | 0.01305 | 0.01445 | 0.01714 | 0.00953 | 0.01332 |
| **51:01** | 0.10450 | 0.12565 | 0.11329 | 0.10333 | 0.08341 | 0.09510 |
| **51:03** | - | - | - | - | - | 0.00027 |
| **51:05** | 0.00017 | - | - | 0.00049 | 0.00048 | - |
| **51:07** | 0.00034 | 0.00131 | 0.00116 | - | 0.00095 | 0.00027 |
| **51:08** | 0.00068 | 0.00196 | 0.00058 | 0.00196 | 0.00048 | 0.00027 |
| **51:09** | 0.00017 | - | - | - | - | - |
| **52:01** | 0.01534 | 0.01273 | 0.01503 | 0.01273 | 0.01382 | 0.01545 |
| **53:01** | 0.00426 | 0.00620 | 0.00405 | 0.00049 | 0.00143 | 0.00506 |
| **53:05** | 0.00017 | - | 0.00058 | - | - | - |
| **54:01** | 0.00034 | - | - | 0.00098 | - | 0.00053 |
| **55:01** | 0.01551 | 0.01305 | 0.01214 | 0.01322 | 0.01239 | 0.01465 |
| **55:02** | - | - | - | 0.00049 | - | - |
| **56:01** | 0.01142 | 0.01403 | 0.01040 | 0.01028 | 0.01525 | 0.00773 |
| **57:01** | 0.02148 | 0.02578 | 0.03064 | 0.02791 | 0.01811 | 0.02664 |
| **57:02** | 0.00034 | 0.00033 | 0.00173 | 0.00147 | - | 0.00027 |
| **57:03** | 0.00068 | 0.00065 | 0.00173 | 0.00049 | 0.00048 | 0.00027 |
| **58:01** | 0.01313 | 0.01044 | 0.01734 | 0.01224 | 0.00953 | 0.00986 |
| **73:01** | 0.00051 | 0.00065 | 0.00058 | 0.00049 | 0.00048 | 0.00053 |
| **TOTAL** | 1 | 1 | 1 | 1 | 1 | 1 |
| **Legend:** | AF - allele frequency | |  |  |  |  |

| **REGION** | **Zagreb**  **(N=2933)**  **AF** | **Dalmatia**  **(N=1532)**  **AF** | **Istria & Primorje**  **(N=865)**  **AF** | **Central Croatia**  **(N=1021)**  **AF** | **North Croatia**  **(N=1049)**  **AF** | **East Croatia**  **(N=1877)**  **AF** |
| --- | --- | --- | --- | --- | --- | --- |
| **HLA-DRB1*** |  |  |  |  |  |  |
| **01:01** | 0.09990 | 0.08094 | 0.09480 | 0.09549 | 0.10724 | 0.09803 |
| **01:02** | 0.01193 | 0.00914 | 0.01098 | 0.01469 | 0.01239 | 0.00879 |
| **01:03** | 0.00119 | 0.00163 | 0.00231 | 0.00098 | 0.00143 | 0.00186 |
| **01:31** | - | - | 0.00058 | - | - | - |
| **03:01** | 0.10058 | 0.11684 | 0.11272 | 0.09892 | 0.10629 | 0.10522 |
| **03:05** | - | - | - | - | - | 0.00027 |
| **03:06** | 0.00017 | - | - | - | - | - |
| **04:01** | 0.02830 | 0.02546 | 0.02428 | 0.02644 | 0.03956 | 0.03010 |
| **04:02** | 0.02523 | 0.02807 | 0.02370 | 0.02644 | 0.01096 | 0.02025 |
| **04:03** | 0.01330 | 0.01501 | 0.00983 | 0.01077 | 0.00715 | 0.01279 |
| **04:04** | 0.01654 | 0.02089 | 0.02254 | 0.01665 | 0.01478 | 0.02104 |
| **04:05** | 0.00170 | 0.00392 | 0.00578 | 0.00441 | 0.00143 | 0.00373 |
| **04:06** | 0.00034 | 0.00033 | 0.00058 | - | 0.00048 | - |
| **04:07** | 0.00324 | 0.00163 | 0.00289 | 0.00637 | 0.00477 | 0.00506 |
| **04:08** | 0.00273 | 0.00131 | 0.00347 | 0.00343 | 0.00334 | 0.00320 |
| **04:15** | 0.00017 | - | - | - | 0.00143 | 0.00027 |
| **07:01** | 0.08848 | 0.07735 | 0.08902 | 0.09892 | 0.08198 | 0.09963 |
| **08:01** | 0.02915 | 0.02578 | 0.02254 | 0.02889 | 0.03003 | 0.02850 |
| **08:02** | 0.00188 | 0.00098 | 0.00231 | 0.00147 | 0.00143 | 0.00133 |
| **08:03** | 0.00222 | - | 0.00231 | - | 0.00095 | 0.00053 |
| **08:04** | 0.00477 | 0.00261 | 0.00289 | 0.00539 | 0.00238 | 0.00160 |
| **09:01** | 0.00256 | 0.00163 | 0.00173 | 0.00147 | 0.00429 | 0.00213 |
| **10:01** | 0.01023 | 0.00718 | 0.01387 | 0.01077 | 0.00858 | 0.01039 |
| **11:01** | 0.07961 | 0.07082 | 0.08092 | 0.09158 | 0.08341 | 0.08205 |
| **11:02** | 0.00188 | 0.00163 | 0.00173 | 0.00245 | 0.00143 | 0.00107 |
| **11:03** | 0.00921 | 0.00946 | 0.00867 | 0.01028 | 0.01192 | 0.00932 |
| **11:04** | 0.07671 | 0.08420 | 0.09422 | 0.07884 | 0.07769 | 0.07725 |
| **11:06** | 0.00034 | 0.00033 | 0.00116 | - | 0.00048 | 0.00053 |
| **11:11** | 0.00051 | 0.00098 | - | 0.00049 | - | - |
| **11:12** | 0.00102 | 0.00131 | 0.00289 | 0.00147 | 0.00048 | 0.00160 |
| **11:15** | 0.00017 | 0.00033 | - | - | - | 0.00027 |
| **11:28** | 0.00017 | 0.00098 | 0.00058 | - | - | - |
| **11:58** | 0.00034 | 0.00033 | - | 0.00049 | - | - |
| **12:01** | 0.01466 | 0.01567 | 0.01792 | 0.01518 | 0.01430 | 0.01518 |
| **12:02** | - | - | 0.00116 | 0.00049 | 0.00048 | - |
| **12:39** | - | - | - | 0.00049 | - | - |
| **13:01** | 0.05967 | 0.07539 | 0.06474 | 0.05436 | 0.05767 | 0.05940 |
| **13:02** | 0.04177 | 0.05385 | 0.04566 | 0.04261 | 0.04051 | 0.03463 |
| **13:03** | 0.00852 | 0.00914 | 0.01040 | 0.00979 | 0.00763 | 0.01412 |
| **13:05** | 0.00375 | 0.00424 | 0.00347 | 0.00196 | 0.00095 | 0.00320 |
| **13:15** | 0.00017 | 0.00065 | - | - | - | 0.00027 |
| **13:32** | - | - | - | - | 0.00095 | - |
| **14:01:01G** | 0.03819 | 0.03101 | 0.03006 | 0.03918 | 0.04337 | 0.04129 |
| **14:02** | - | - | - | - | 0.00048 | - |
| **14:04** | 0.00051 | 0.00098 | - | 0.00147 | - | 0.00240 |
| **14:05** | 0.00034 | - | - | 0.00098 | - | 0.00053 |
| **14:07** | 0.00017 | 0.00065 | - | - | - | - |
| **15:01** | 0.09018 | 0.09432 | 0.09422 | 0.08227 | 0.07388 | 0.08657 |
| **15:02** | 0.01415 | 0.01240 | 0.01445 | 0.01126 | 0.01001 | 0.01492 |
| **15:03** | 0.00188 | - | 0.00058 | 0.00147 | 0.00095 | 0.00107 |
| **15:07** | 0.00017 | - | - | - | 0.00048 | - |
| **16:01** | 0.10365 | 0.10313 | 0.07225 | 0.09305 | 0.13012 | 0.09217 |
| **16:02** | 0.00767 | 0.00751 | 0.00578 | 0.00833 | 0.00238 | 0.00746 |
| **TOTAL** | 1 | 1 | 1 | 1 | 1 | 1 |
| **Legend:** | AF - allele frequency | |  |  |  |  |
