# Supplementary material for: Mapping the Human Leukocyte Antigen Diversity among Croatian Regions: Implication in Transplantation
Source: J Immunol Res. 2021 Apr 7;2021:6670960. doi: 10.1155/2021/6670960 (PMC8051524; doi:10.1155/2021/6670960)
Supplement: Supplementary 3 — sTable 3: the distribution of HLA haplotypes in Zagreb area and five Croatian regions. [file 6670960.f3.doc]

**s Table 3. The distribution of HLA-A~B~C~**DRB1 haplotypes in Zagreb area and five Croatian regions

| **Zagreb (N=2933)** |  |  |
| --- | --- | --- |
| **Haplotypes HLA-A*~C*~B*~DRB1* sorted by frequency** | **frequency** | **copies** |
| 01:01~07:01~08:01~03:01 | 0.04655 | 273.1 |
| 03:01~07:02~07:02~15:01 | 0.01671 | 98.0 |
| 02:01~07:01~18:01~11:04 | 0.01499 | 87.9 |
| 02:01~02:02~27:02~16:01 | 0.01170 | 68.6 |
| 11:01~04:01~35:01~01:01 | 0.01109 | 65.0 |
| 02:01~07:04~44:27~16:01 | 0.01060 | 62.2 |
| 03:01~04:01~35:01~01:01 | 0.01060 | 62.2 |
| 02:01~06:02~13:02~07:01 | 0.00984 | 57.7 |
| 23:01~04:01~44:03~07:01 | 0.00940 | 55.2 |
| 02:01~02:02~27:05~01:01 | 0.00860 | 50.4 |
| 26:01~12:03~38:01~04:02 | 0.00744 | 43.6 |
| 25:01~12:03~18:01~15:01 | 0.00728 | 42.7 |
| 33:01~08:02~14:02~01:02 | 0.00645 | 37.8 |
| 02:01~07:02~07:02~15:01 | 0.00601 | 35.2 |
| 02:01~06:02~57:01~07:01 | 0.00552 | 32.4 |
| 02:01~07:01~08:01~03:01 | 0.00474 | 27.8 |
| 01:01~12:02~52:01~15:02 | 0.00454 | 26.6 |
| 33:01~08:02~14:02~03:01 | 0.00453 | 26.6 |
| 02:01~05:01~44:02~04:01 | 0.00429 | 25.2 |
| 24:02~04:01~35:02~11:04 | 0.00381 | 22.4 |
| 02:01~03:04~15:01~04:01 | 0.00378 | 22.2 |
| 30:01~06:02~13:02~07:01 | 0.00374 | 22.0 |
| 02:01~01:02~51:01~04:02 | 0.00370 | 21.7 |
| 02:01~12:03~38:01~13:01 | 0.00364 | 21.3 |
| 24:02~06:02~13:02~07:01 | 0.00348 | 20.4 |
| 02:01~06:02~57:01~16:01 | 0.00348 | 20.4 |
| 02:01~04:01~35:01~14:01:01g | 0.00345 | 20.2 |
| 24:02~15:02~51:01~11:01 | 0.00336 | 19.7 |
| 02:01~14:02~51:01~16:01 | 0.00333 | 19.5 |
| 03:01~07:01~18:01~11:04 | 0.00331 | 19.4 |
| 02:01~02:02~44:05~16:01 | 0.00322 | 18.9 |
| 02:01~12:03~18:01~16:01 | 0.00319 | 18.7 |
| 03:01~04:01~35:03~04:03 | 0.00318 | 18.7 |
| 11:01~07:01~18:01~11:04 | 0.00317 | 18.6 |
| 02:01~04:01~35:03~16:01 | 0.00313 | 18.3 |
| 26:01~12:03~38:01~13:01 | 0.00304 | 17.9 |
| 68:01~04:01~35:03~08:01 | 0.00302 | 17.7 |
| 11:01~07:02~07:02~15:01 | 0.00292 | 17.1 |
| 02:01~04:01~35:03~11:01 | 0.00281 | 16.5 |
| 03:01~07:02~07:02~16:01 | 0.00281 | 16.5 |
| 11:01~12:02~52:01~01:01 | 0.00271 | 15.9 |
| 03:01~07:02~07:02~11:01 | 0.00258 | 15.2 |
| 02:01~02:02~51:01~11:01 | 0.00257 | 15.1 |
| 24:02~07:02~07:02~15:01 | 0.00255 | 15.0 |
| 01:01~06:02~57:01~07:01 | 0.00254 | 14.9 |
| 03:01~02:02~27:02~16:01 | 0.00254 | 14.9 |
| 01:01~06:02~37:01~15:01 | 0.00253 | 14.8 |
| 03:01~07:02~07:02~13:02 | 0.00250 | 14.6 |
| 01:01~07:01~18:01~11:04 | 0.00248 | 14.6 |
| 02:01~03:03~15:01~13:01 | 0.00244 | 14.3 |
| 24:02~07:01~18:01~11:04 | 0.00244 | 14.3 |
| 02:01~14:02~51:01~08:01 | 0.00243 | 14.2 |
| 02:01~05:01~44:02~16:01 | 0.00240 | 14.1 |
| 24:02~07:02~07:02~01:01 | 0.00238 | 14.0 |
| 02:01~05:01~44:02~13:01 | 0.00234 | 13.7 |
| 01:01~06:02~13:02~07:01 | 0.00232 | 13.6 |
| 02:01~14:02~51:01~01:01 | 0.00228 | 13.4 |
| 24:02~01:02~51:01~11:01 | 0.00226 | 13.2 |
| 01:01~07:01~58:01~08:04 | 0.00221 | 13.0 |
| 29:02~16:01~44:03~07:01 | 0.00220 | 12.9 |
| 31:01~04:01~35:03~03:01 | 0.00218 | 12.8 |
| 68:01~02:02~27:02~16:01 | 0.00217 | 12.7 |
| 24:02~07:01~08:01~03:01 | 0.00216 | 12.7 |
| 02:01~04:01~44:03~07:01 | 0.00216 | 12.6 |
| 24:02~12:03~39:01~16:01 | 0.00215 | 12.6 |
| 02:01~12:03~38:01~04:02 | 0.00214 | 12.6 |
| 02:01~02:02~40:02~11:01 | 0.00208 | 12.2 |
| 33:01~03:02~58:01~13:02 | 0.00207 | 12.2 |
| 32:01~04:01~35:01~01:01 | 0.00206 | 12.1 |
| 03:01~07:04~44:27~16:01 | 0.00206 | 12.1 |
| 32:01~12:03~51:01~01:01 | 0.00203 | 11.9 |
| 02:01~15:02~51:01~07:01 | 0.00200 | 11.7 |
| 11:01~04:01~35:01~16:01 | 0.00198 | 11.6 |
| 02:01~01:02~51:01~14:01:01g | 0.00197 | 11.5 |
| 03:01~04:01~35:03~13:02 | 0.00191 | 11.2 |
| 02:01~04:01~35:03~11:04 | 0.00190 | 11.1 |
| 24:02~12:03~38:01~13:01 | 0.00188 | 11.1 |
| 02:01~17:03~41:02~13:03 | 0.00188 | 11.0 |
| 01:01~07:02~07:02~15:01 | 0.00185 | 10.8 |
| 26:01~01:02~27:05~01:01 | 0.00184 | 10.8 |
| 02:01~07:01~49:01~13:02 | 0.00182 | 10.7 |
| 26:01~12:03~38:01~16:01 | 0.00182 | 10.7 |
| 02:01~04:01~15:01~01:01 | 0.00180 | 10.5 |
| 02:01~12:03~38:01~11:04 | 0.00178 | 10.5 |
| 01:01~07:01~08:01~07:01 | 0.00177 | 10.4 |
| 03:01~07:02~07:02~01:01 | 0.00177 | 10.4 |
| 02:01~02:02~27:05~15:01 | 0.00174 | 10.2 |
| 24:02~02:02~27:05~13:01 | 0.00171 | 10.0 |
| 32:01~12:03~38:01~15:01 | 0.00171 | 10.0 |
| 68:02~04:01~53:01~13:02 | 0.00170 | 10.0 |
| 02:05~06:02~50:01~07:01 | 0.00170 | 10.0 |
| 03:01~08:02~14:02~01:02 | 0.00169 | 9.9 |
| 24:02~03:03~15:01~04:02 | 0.00169 | 9.9 |
| 24:02~02:02~51:01~11:04 | 0.00168 | 9.9 |
| 02:01~12:03~18:01~01:01 | 0.00167 | 9.8 |
| 02:01~03:04~40:01~13:02 | 0.00167 | 9.8 |
| 02:01~03:03~15:01~04:01 | 0.00165 | 9.7 |
| 02:01~01:02~56:01~01:01 | 0.00165 | 9.7 |
| 02:01~07:01~18:01~14:01:01g | 0.00164 | 9.6 |
| 02:01~01:02~51:01~11:01 | 0.00163 | 9.6 |
| 23:01~07:01~49:01~11:01 | 0.00161 | 9.5 |
| 01:01~07:01~49:01~13:02 | 0.00161 | 9.5 |
| 68:01~12:03~39:01~16:01 | 0.00161 | 9.4 |
| 02:01~12:03~38:01~15:01 | 0.00161 | 9.4 |
| 11:01~04:01~35:01~13:01 | 0.00160 | 9.4 |
| 02:01~14:02~51:01~13:01 | 0.00154 | 9.0 |
| 01:01~12:03~51:01~11:04 | 0.00153 | 9.0 |
| 30:04~08:02~14:01~04:04 | 0.00153 | 9.0 |
| 32:01~05:01~44:02~04:01 | 0.00153 | 9.0 |
| 02:01~01:02~27:05~01:01 | 0.00153 | 9.0 |
| 02:01~02:02~40:02~13:01 | 0.00150 | 8.8 |
| 02:01~12:03~39:01~11:01 | 0.00148 | 8.7 |
| 26:01~07:02~07:02~15:01 | 0.00147 | 8.6 |
| 03:01~12:03~35:03~14:01G | 0.00146 | 8.6 |
| 03:01~07:02~07:02~07:01 | 0.00146 | 8.5 |
| 32:01~04:01~35:01~16:01 | 0.00144 | 8.5 |
| 02:01~05:01~44:02~11:04 | 0.00144 | 8.5 |
| 24:02~07:01~18:01~01:01 | 0.00143 | 8.4 |
| 02:01~12:02~52:01~15:02 | 0.00143 | 8.4 |
| 01:01~07:01~08:01~01:01 | 0.00142 | 8.4 |
| 24:02~04:01~35:01~01:01 | 0.00142 | 8.3 |
| 02:01~04:01~35:01~01:01 | 0.00137 | 8.0 |
| 02:01~03:04~40:01~04:04 | 0.00136 | 8.0 |
| 32:01~02:02~40:02~16:01 | 0.00136 | 8.0 |
| 26:01~12:03~38:01~14:01:01g | 0.00135 | 7.9 |
| 24:02~03:03~15:01~13:01 | 0.00135 | 7.9 |
| 24:02~12:03~18:01~11:04 | 0.00134 | 7.9 |
| 25:01~12:03~18:01~04:01 | 0.00133 | 7.8 |
| 24:02~04:01~35:01~07:01 | 0.00130 | 7.6 |
| 03:01~07:02~07:02~03:01 | 0.00129 | 7.6 |
| 24:02~14:02~51:01~13:01 | 0.00128 | 7.5 |
| 01:01~07:02~08:01~03:01 | 0.00128 | 7.5 |
| 02:01~06:02~50:01~07:01 | 0.00127 | 7.4 |
| 03:01~04:01~35:01~04:01 | 0.00125 | 7.3 |
| 01:01~04:01~35:01~14:01:01g | 0.00124 | 7.3 |
| 02:01~01:02~51:01~16:01 | 0.00124 | 7.3 |
| 02:01~12:03~35:03~14:01:01g | 0.00124 | 7.3 |
| 02:01~15:02~51:01~11:01 | 0.00124 | 7.3 |
| 24:02~02:02~27:02~16:01 | 0.00123 | 7.2 |
| 68:01~15:02~51:01~13:01 | 0.00123 | 7.2 |
| 24:02~05:01~44:02~16:01 | 0.00123 | 7.2 |
| 24:02~04:01~35:01~13:01 | 0.00123 | 7.2 |
| 02:01~12:03~39:01~12:01 | 0.00122 | 7.2 |
| 02:01~07:01~49:01~11:01 | 0.00122 | 7.1 |
| 24:02~12:03~18:01~03:01 | 0.00121 | 7.1 |
| 03:01~15:02~51:01~13:02 | 0.00121 | 7.1 |
| 25:01~07:02~07:02~03:01 | 0.00120 | 7.1 |
| 11:01~03:03~55:01~15:01 | 0.00120 | 7.0 |
| 02:01~06:02~50:01~03:01 | 0.00120 | 7.0 |
| 23:01~07:02~07:02~15:03 | 0.00119 | 7.0 |
| 68:01~07:01~18:01~16:01 | 0.00118 | 6.9 |
| 32:01~02:02~40:02~16:02 | 0.00117 | 6.9 |
| 03:01~03:04~40:01~13:02 | 0.00117 | 6.9 |
| 25:01~12:03~18:01~01:01 | 0.00117 | 6.8 |
| 26:01~01:02~51:01~11:04 | 0.00116 | 6.8 |
| 68:01~04:01~35:03~11:01 | 0.00115 | 6.7 |
| 02:01~04:01~35:01~11:01 | 0.00113 | 6.7 |
| 11:01~12:02~52:01~15:02 | 0.00113 | 6.6 |
| 03:01~04:01~35:03~16:01 | 0.00113 | 6.6 |
| 02:01~04:01~35:01~03:01 | 0.00111 | 6.5 |
| 02:01~07:02~07:02~08:01 | 0.00111 | 6.5 |
| 02:01~12:03~39:01~16:01 | 0.00111 | 6.5 |
| 02:01~12:03~18:01~15:01 | 0.00111 | 6.5 |
| 03:01~04:01~35:03~15:01 | 0.00110 | 6.4 |
| 68:01~04:01~35:03~15:01 | 0.00110 | 6.4 |
| 24:02~02:02~44:05~01:01 | 0.00108 | 6.4 |
| 11:01~02:02~40:02~11:01 | 0.00107 | 6.3 |
| 02:01~03:03~35:01~08:01 | 0.00106 | 6.2 |
| 03:01~12:03~51:01~01:01 | 0.00106 | 6.2 |
| 24:02~05:01~44:02~13:01 | 0.00105 | 6.1 |
| 01:01~06:02~37:01~07:01 | 0.00103 | 6.0 |
| 02:01~07:01~18:01~11:01 | 0.00103 | 6.0 |
| 32:01~03:03~15:01~13:01 | 0.00102 | 6.0 |
| 31:01~03:04~40:01~04:04 | 0.00102 | 6.0 |
| 24:02~15:02~51:01~04:01 | 0.00102 | 6.0 |
| 26:01~01:02~56:01~08:01 | 0.00102 | 6.0 |
| 32:01~17:01~41:01~03:01 | 0.00102 | 6.0 |
| 25:01~14:02~51:01~04:07 | 0.00102 | 6.0 |
| 11:01~15:02~51:01~04:04 | 0.00102 | 6.0 |
| 24:02~04:01~44:03~11:01 | 0.00101 | 6.0 |
| 24:02~06:02~13:02~10:01 | 0.00101 | 5.9 |
| 01:01~02:02~40:02~16:02 | 0.00101 | 5.9 |
| 03:01~12:03~38:01~13:01 | 0.00100 | 5.9 |
| 25:01~07:01~08:01~03:01 | 0.00100 | 5.9 |
| 02:01~07:04~44:27~14:01:01g | 0.00100 | 5.9 |
| 11:01~03:03~55:01~14:01:01g | 0.00099 | 5.8 |
| 03:01~06:02~13:02~07:01 | 0.00099 | 5.8 |
| 24:02~07:02~07:02~16:01 | 0.00098 | 5.8 |
| 01:01~01:02~56:01~08:01 | 0.00098 | 5.7 |
| 03:01~04:01~35:01~14:01G | 0.00098 | 5.7 |
| 68:01~07:04~44:27~11:01 | 0.00098 | 5.7 |
| 01:01~06:02~37:01~10:01 | 0.00097 | 5.7 |
| 68:01~12:03~38:01~15:01 | 0.00097 | 5.7 |
| 03:01~15:02~51:01~11:01 | 0.00096 | 5.6 |
| 26:01~12:03~38:01~01:01 | 0.00095 | 5.6 |
| 02:01~12:03~38:01~16:01 | 0.00095 | 5.6 |
| 03:01~04:01~35:03~13:01 | 0.00095 | 5.6 |
| 24:02~05:01~44:02~15:01 | 0.00095 | 5.6 |
| 24:02~02:02~44:05~11:01 | 0.00095 | 5.5 |
| 02:01~14:02~51:01~11:04 | 0.00094 | 5.5 |
| 02:01~05:01~44:02~01:01 | 0.00094 | 5.5 |
| 01:01~04:01~35:01~01:01 | 0.00094 | 5.5 |
| 02:01~15:02~51:01~13:02 | 0.00093 | 5.4 |
| 11:01~04:01~35:01~11:01 | 0.00093 | 5.4 |
| 01:01~07:02~07:02~11:01 | 0.00092 | 5.4 |
| 02:01~06:02~13:02~11:01 | 0.00092 | 5.4 |
| 02:01~05:01~44:02~14:01:01g | 0.00091 | 5.4 |
| 32:01~04:01~35:01~14:01G | 0.00091 | 5.3 |
| 32:01~02:02~40:02~11:01 | 0.00091 | 5.3 |
| 24:02~04:01~35:03~13:01 | 0.00091 | 5.3 |
| 02:01~12:03~18:01~11:04 | 0.00091 | 5.3 |
| 31:01~05:01~44:02~11:04 | 0.00091 | 5.3 |
| 02:01~04:01~35:02~11:04 | 0.00090 | 5.3 |
| 02:01~04:01~35:03~13:02 | 0.00089 | 5.2 |
| 01:01~07:01~08:01~15:01 | 0.00089 | 5.2 |
| 03:01~04:01~35:01~11:04 | 0.00088 | 5.2 |
| 02:01~07:02~07:02~14:01:01g | 0.00088 | 5.2 |
| 02:01~07:01~18:01~13:01 | 0.00088 | 5.2 |
| 02:01~04:01~35:01~13:01 | 0.00088 | 5.1 |
| 02:01~07:01~18:01~04:01 | 0.00088 | 5.1 |
| 02:01~02:02~27:05~11:01 | 0.00088 | 5.1 |
| 02:01~01:02~56:01~08:01 | 0.00087 | 5.1 |
| 24:02~02:02~27:05~11:01 | 0.00087 | 5.1 |
| 02:01~03:03~15:01~16:01 | 0.00087 | 5.1 |
| 24:02~03:03~15:01~01:01 | 0.00086 | 5.0 |
| 01:01~07:01~08:01~16:01 | 0.00086 | 5.0 |
| 25:01~06:02~13:02~07:01 | 0.00086 | 5.0 |
| 02:01~06:02~13:02~16:01 | 0.00085 | 5.0 |
| 26:01~03:03~55:01~11:04 | 0.00085 | 5.0 |
| 24:02~01:02~56:01~11:04 | 0.00085 | 5.0 |
| 24:02~16:04~44:02~15:01 | 0.00085 | 5.0 |
| 03:01~12:03~51:01~11:04 | 0.00085 | 5.0 |
| 24:02~07:01~18:01~14:01:01g | 0.00085 | 5.0 |
| 66:01~08:02~14:01~07:01 | 0.00085 | 5.0 |
| 01:01~02:02~27:02~16:02 | 0.00085 | 5.0 |
| 33:03~03:02~58:01~03:01 | 0.00085 | 5.0 |
| 03:01~05:01~27:02~11:03 | 0.00085 | 5.0 |
| 68:02~08:02~14:02~13:03 | 0.00085 | 5.0 |
| 02:05~07:01~49:01~07:01 | 0.00085 | 5.0 |
| 25:01~03:03~15:01~04:03 | 0.00085 | 5.0 |
| 24:02~06:02~50:01~11:01 | 0.00084 | 4.9 |
| 32:01~03:03~15:01~11:01 | 0.00084 | 4.9 |
| 68:01~07:02~07:02~13:02 | 0.00084 | 4.9 |
| 02:01~15:02~51:01~15:01 | 0.00084 | 4.9 |
| 03:01~08:02~14:02~11:01 | 0.00084 | 4.9 |
| 02:01~05:01~44:02~12:01 | 0.00084 | 4.9 |
| 01:01~04:01~44:03~07:01 | 0.00084 | 4.9 |
| 03:01~07:02~07:02~14:01:01g | 0.00084 | 4.9 |
| 02:01~15:02~51:01~13:01 | 0.00083 | 4.9 |
| 68:01~14:02~51:01~11:01 | 0.00083 | 4.8 |
| 03:01~06:02~13:02~11:01 | 0.00082 | 4.8 |
| 02:01~16:02~44:03~07:01 | 0.00082 | 4.8 |
| 01:01~07:01~49:01~03:01 | 0.00082 | 4.8 |
| 32:01~12:03~39:01~16:01 | 0.00081 | 4.8 |
| 03:01~03:04~15:01~04:01 | 0.00081 | 4.7 |
| 03:01~04:01~35:01~15:01 | 0.00080 | 4.7 |
| 03:01~02:02~40:02~11:01 | 0.00078 | 4.6 |
| 26:01~07:01~49:01~13:02 | 0.00078 | 4.6 |
| 01:01~07:01~08:01~11:01 | 0.00077 | 4.5 |
| 26:01~12:03~39:01~16:01 | 0.00077 | 4.5 |
| 68:01~04:01~35:03~01:01 | 0.00076 | 4.4 |
| 02:01~07:04~44:27~13:01 | 0.00075 | 4.4 |
| 02:01~05:01~44:02~15:01 | 0.00075 | 4.4 |
| 02:01~03:02~58:01~13:02 | 0.00075 | 4.4 |
| 01:01~07:01~08:01~13:01 | 0.00075 | 4.4 |
| 01:01~06:02~35:02~11:04 | 0.00074 | 4.4 |
| 02:01~07:02~07:02~07:01 | 0.00074 | 4.3 |
| 23:01~06:02~50:01~03:01 | 0.00074 | 4.3 |
| 26:01~07:01~49:01~01:01 | 0.00073 | 4.3 |
| 11:01~01:02~56:01~01:01 | 0.00072 | 4.2 |
| 02:01~03:03~15:01~16:02 | 0.00072 | 4.2 |
| 24:02~04:01~44:03~07:01 | 0.00072 | 4.2 |
| 02:01~07:02~07:02~03:01 | 0.00072 | 4.2 |
| 24:02~07:01~18:01~11:01 | 0.00072 | 4.2 |
| 11:01~12:03~38:01~14:01:01g | 0.00072 | 4.2 |
| 24:02~05:01~44:02~07:01 | 0.00072 | 4.2 |
| 24:02~01:02~51:01~01:01 | 0.00072 | 4.2 |
| 02:01~03:03~15:01~13:02 | 0.00071 | 4.2 |
| 01:01~15:02~51:01~15:01 | 0.00070 | 4.1 |
| 02:01~01:02~56:01~15:01 | 0.00070 | 4.1 |
| 02:01~14:02~51:01~11:01 | 0.00070 | 4.1 |
| 25:01~06:02~57:01~07:01 | 0.00070 | 4.1 |
| 02:01~02:02~27:05~08:01 | 0.00070 | 4.1 |
| 24:02~12:03~35:03~14:01:01g | 0.00070 | 4.1 |
| 02:01~04:01~35:08~03:01 | 0.00070 | 4.1 |
| 68:01~07:01~18:01~13:01 | 0.00069 | 4.1 |
| 03:01~08:02~14:02~15:01 | 0.00069 | 4.1 |
| 03:01~01:02~51:01~01:01 | 0.00069 | 4.1 |
| 33:01~08:02~14:02~11:04 | 0.00069 | 4.1 |
| 03:01~03:04~15:01~01:01 | 0.00069 | 4.0 |
| 24:02~03:03~55:01~01:01 | 0.00069 | 4.0 |
| 01:01~04:01~35:02~11:04 | 0.00068 | 4.0 |
| 11:01~02:02~27:05~11:01 | 0.00068 | 4.0 |
| 02:01~05:01~44:02~07:01 | 0.00068 | 4.0 |
| 32:01~04:01~35:03~01:01 | 0.00068 | 4.0 |
| 24:02~01:02~27:05~01:01 | 0.00068 | 4.0 |
| 25:01~12:03~18:01~14:01:01g | 0.00068 | 4.0 |
| 26:01~03:03~55:01~14:01:01g | 0.00068 | 4.0 |
| 30:01~06:02~13:02~08:02 | 0.00068 | 4.0 |
| 24:02~03:03~55:01~16:01 | 0.00068 | 4.0 |
| 68:01~04:01~35:03~11:12 | 0.00068 | 4.0 |
| 32:01~02:02~27:05~15:01 | 0.00068 | 4.0 |
| 02:01~16:02~51:01~03:01 | 0.00068 | 4.0 |
| 24:02~04:01~35:01~12:01 | 0.00068 | 4.0 |
| 26:01~12:03~18:01~11:04 | 0.00068 | 4.0 |
| 03:01~12:03~39:01~13:01 | 0.00068 | 4.0 |
| 31:01~07:01~15:17~13:02 | 0.00068 | 4.0 |
| 68:01~01:02~27:05~13:01 | 0.00068 | 4.0 |
| 66:01~17:03~41:02~13:03 | 0.00068 | 4.0 |
| 23:01~12:03~18:01~11:01 | 0.00068 | 4.0 |
| 03:01~04:01~35:03~04:01 | 0.00068 | 4.0 |
| 03:01~12:03~39:01~14:01:01g | 0.00068 | 4.0 |
| 02:01~03:04~40:01~04:01 | 0.00068 | 4.0 |
| 11:01~12:03~07:02~15:01 | 0.00068 | 4.0 |
| 02:01~07:01~18:01~15:01 | 0.00068 | 4.0 |
| 25:01~12:03~18:01~03:01 | 0.00068 | 4.0 |
| 11:01~06:02~13:02~07:01 | 0.00068 | 4.0 |
| 26:01~06:02~57:01~15:01 | 0.00068 | 4.0 |
| 24:02~02:02~27:05~01:01 | 0.00068 | 4.0 |
| 31:01~12:03~39:01~12:01 | 0.00068 | 4.0 |

| **Dalmatia (N=1532)** |  |  |
| --- | --- | --- |
| **Haplotypes HLA-A*~C*~B*~DRB1* sorted by frequency** | **frequency** | **copies** |
| 01:01~07:01~08:01~03:01 | 0.06405 | 196.3 |
| 03:01~07:02~07:02~15:01 | 0.01341 | 41.1 |
| 02:01~07:01~18:01~11:04 | 0.01251 | 38.3 |
| 11:01~07:01~18:01~11:04 | 0.00972 | 29.8 |
| 24:02~07:02~07:02~15:01 | 0.00935 | 28.7 |
| 02:01~06:02~13:02~07:01 | 0.00909 | 27.8 |
| 11:01~04:01~35:01~01:01 | 0.00863 | 26.4 |
| 02:01~02:02~27:05~01:01 | 0.00849 | 26.0 |
| 26:01~12:03~38:01~04:02 | 0.00844 | 25.8 |
| 25:01~12:03~18:01~15:01 | 0.00818 | 25.0 |
| 02:01~02:02~27:02~16:01 | 0.00735 | 22.5 |
| 02:01~06:02~57:01~07:01 | 0.00710 | 21.7 |
| 02:01~12:03~18:01~16:01 | 0.00679 | 20.8 |
| 68:01~12:03~39:01~16:01 | 0.00651 | 20.0 |
| 23:01~04:01~44:03~07:01 | 0.00651 | 19.9 |
| 02:01~07:04~44:27~16:01 | 0.00642 | 19.7 |
| 02:01~01:02~51:01~04:02 | 0.00618 | 18.9 |
| 02:01~14:02~51:01~16:01 | 0.00587 | 18.0 |
| 03:01~07:01~18:01~11:04 | 0.00586 | 18.0 |
| 02:01~06:02~57:01~16:01 | 0.00554 | 17.0 |
| 03:01~04:01~35:01~01:01 | 0.00553 | 16.9 |
| 02:01~05:01~44:02~04:01 | 0.00550 | 16.9 |
| 01:01~07:01~49:01~13:02 | 0.00491 | 15.0 |
| 02:01~12:03~38:01~13:01 | 0.00474 | 14.5 |
| 02:01~07:01~08:01~03:01 | 0.00462 | 14.1 |
| 02:01~07:02~07:02~15:01 | 0.00451 | 13.8 |
| 26:01~12:03~38:01~13:01 | 0.00427 | 13.1 |
| 33:01~08:02~14:02~01:02 | 0.00424 | 13.0 |
| 24:02~06:02~13:02~07:01 | 0.00415 | 12.7 |
| 03:01~04:01~35:03~01:01 | 0.00414 | 12.7 |
| 02:01~12:03~39:01~16:01 | 0.00409 | 12.5 |
| 02:01~01:02~51:01~14:01:01g | 0.00400 | 12.2 |
| 68:02~04:01~53:01~13:02 | 0.00392 | 12.0 |
| 32:01~02:02~40:02~16:02 | 0.00392 | 12.0 |
| 11:01~07:02~07:02~15:01 | 0.00390 | 11.9 |
| 02:01~02:02~40:02~11:01 | 0.00385 | 11.8 |
| 02:01~04:01~35:03~11:04 | 0.00384 | 11.8 |
| 03:01~07:02~07:02~11:01 | 0.00376 | 11.5 |
| 24:02~14:02~51:01~13:01 | 0.00359 | 11.0 |
| 30:04~08:02~14:01~04:04 | 0.00359 | 11.0 |
| 24:02~04:01~35:02~11:04 | 0.00359 | 11.0 |
| 03:01~04:01~35:03~04:03 | 0.00355 | 10.9 |
| 02:01~15:02~51:01~01:01 | 0.00343 | 10.5 |
| 01:01~07:01~18:01~11:04 | 0.00336 | 10.3 |
| 01:01~12:02~52:01~15:02 | 0.00329 | 10.1 |
| 24:02~02:02~27:05~13:01 | 0.00326 | 10.0 |
| 02:01~14:02~51:01~08:01 | 0.00326 | 10.0 |
| 68:01~04:01~35:03~08:01 | 0.00325 | 10.0 |
| 02:01~15:02~51:01~08:01 | 0.00319 | 9.8 |
| 11:01~07:01~08:01~03:01 | 0.00307 | 9.4 |
| 02:01~12:03~18:01~01:01 | 0.00301 | 9.2 |
| 02:05~06:02~50:01~07:01 | 0.00299 | 9.1 |
| 33:01~08:02~14:02~03:01 | 0.00294 | 9.0 |
| 03:01~08:02~14:02~01:02 | 0.00294 | 9.0 |
| 02:01~04:01~15:01~01:01 | 0.00294 | 9.0 |
| 02:01~17:03~41:02~13:03 | 0.00294 | 9.0 |
| 02:01~12:03~38:01~04:02 | 0.00291 | 8.9 |
| 03:02~04:01~35:03~13:02 | 0.00289 | 8.9 |
| 11:01~12:03~39:01~16:01 | 0.00287 | 8.8 |
| 32:01~12:03~38:01~15:01 | 0.00287 | 8.8 |
| 32:01~04:01~35:01~16:01 | 0.00287 | 8.8 |
| 11:01~15:02~51:01~04:04 | 0.00284 | 8.7 |
| 02:01~12:02~52:01~15:02 | 0.00275 | 8.4 |
| 03:01~07:04~44:27~16:01 | 0.00271 | 8.3 |
| 24:02~04:01~35:03~13:01 | 0.00270 | 8.3 |
| 24:02~12:03~38:01~13:01 | 0.00270 | 8.3 |
| 32:01~03:03~15:01~11:01 | 0.00264 | 8.1 |
| 02:01~07:01~18:01~15:01 | 0.00263 | 8.1 |
| 02:01~02:02~51:01~11:01 | 0.00261 | 8.0 |
| 02:01~02:02~44:05~16:01 | 0.00261 | 8.0 |
| 26:01~12:03~18:01~11:04 | 0.00261 | 8.0 |
| 24:02~05:01~44:02~07:01 | 0.00261 | 8.0 |
| 68:01~15:02~51:01~13:01 | 0.00261 | 8.0 |
| 30:01~06:02~13:02~07:01 | 0.00261 | 8.0 |
| 02:01~07:01~49:01~11:01 | 0.00255 | 7.8 |
| 02:01~03:03~15:01~13:01 | 0.00254 | 7.8 |
| 02:01~14:02~51:01~11:01 | 0.00252 | 7.7 |
| 02:01~05:01~44:02~13:01 | 0.00252 | 7.7 |
| 01:01~07:02~07:02~15:01 | 0.00250 | 7.7 |
| 02:01~12:03~38:01~16:01 | 0.00248 | 7.6 |
| 24:02~15:02~51:01~11:01 | 0.00241 | 7.4 |
| 01:01~06:02~57:01~07:01 | 0.00230 | 7.0 |
| 26:01~01:02~51:01~11:04 | 0.00228 | 7.0 |
| 11:01~12:03~38:01~04:02 | 0.00228 | 7.0 |
| 24:02~03:03~55:01~16:01 | 0.00228 | 7.0 |
| 11:01~12:03~07:02~15:01 | 0.00227 | 7.0 |
| 32:01~03:03~15:01~01:01 | 0.00226 | 6.9 |
| 68:01~04:01~35:03~15:01 | 0.00225 | 6.9 |
| 68:01~04:01~35:03~11:01 | 0.00219 | 6.7 |
| 11:01~12:02~52:01~15:02 | 0.00212 | 6.5 |
| 01:01~06:02~13:02~07:01 | 0.00203 | 6.2 |
| 02:01~05:01~44:02~01:01 | 0.00200 | 6.1 |
| 02:01~06:02~35:02~11:04 | 0.00199 | 6.1 |
| 02:01~04:01~35:03~16:01 | 0.00196 | 6.0 |
| 26:01~12:03~38:01~14:01G | 0.00196 | 6.0 |
| 02:01~04:01~35:01~11:04 | 0.00196 | 6.0 |
| 01:01~02:02~27:05~03:01 | 0.00196 | 6.0 |
| 02:01~05:01~44:02~15:01 | 0.00196 | 6.0 |
| 24:02~12:03~35:03~01:01 | 0.00196 | 6.0 |
| 03:01~01:02~51:01~11:01 | 0.00196 | 6.0 |
| 02:01~01:02~56:01~08:01 | 0.00196 | 6.0 |
| 02:01~05:01~44:02~07:01 | 0.00195 | 6.0 |
| 01:01~06:02~35:02~11:04 | 0.00193 | 5.9 |
| 02:01~02:02~27:05~15:01 | 0.00183 | 5.6 |
| 02:01~07:01~18:01~11:01 | 0.00180 | 5.5 |
| 02:01~07:01~18:01~16:01 | 0.00180 | 5.5 |
| 02:01~15:02~51:01~07:01 | 0.00179 | 5.5 |
| 24:02~07:01~18:01~11:04 | 0.00173 | 5.3 |
| 01:01~07:01~08:01~13:01 | 0.00173 | 5.3 |
| 01:01~04:01~35:01~04:04 | 0.00172 | 5.3 |
| 11:01~04:01~35:01~16:01 | 0.00170 | 5.2 |
| 03:01~02:02~27:02~16:01 | 0.00170 | 5.2 |
| 02:01~12:03~38:01~11:04 | 0.00169 | 5.2 |
| 11:01~04:01~35:01~11:01 | 0.00168 | 5.1 |
| 03:01~04:01~35:03~13:01 | 0.00168 | 5.1 |
| 02:01~15:02~51:01~16:01 | 0.00167 | 5.1 |
| 02:01~02:02~27:05~13:01 | 0.00165 | 5.0 |
| 01:01~07:01~08:01~13:02 | 0.00165 | 5.0 |
| 68:01~12:03~38:01~15:01 | 0.00164 | 5.0 |
| 03:01~07:02~07:02~07:01 | 0.00163 | 5.0 |
| 24:02~03:03~15:01~11:03 | 0.00163 | 5.0 |
| 01:01~06:02~37:01~15:01 | 0.00163 | 5.0 |
| 31:01~07:01~15:17~13:02 | 0.00163 | 5.0 |
| 11:01~01:02~56:01~01:01 | 0.00163 | 5.0 |
| 32:01~02:02~40:02~11:01 | 0.00163 | 5.0 |
| 24:02~01:02~51:01~04:03 | 0.00163 | 5.0 |
| 33:01~03:02~58:01~13:02 | 0.00163 | 5.0 |
| 30:02~05:01~18:01~03:01 | 0.00163 | 5.0 |
| 03:01~03:04~40:01~13:01 | 0.00163 | 5.0 |
| 30:02~08:02~14:02~11:03 | 0.00163 | 5.0 |
| 31:01~05:01~44:02~11:04 | 0.00163 | 5.0 |
| 32:01~02:02~40:02~16:01 | 0.00163 | 5.0 |
| 03:01~08:02~14:02~11:01 | 0.00163 | 5.0 |
| 26:01~03:03~55:01~11:01 | 0.00162 | 5.0 |
| 02:01~12:03~35:03~14:01:01g | 0.00162 | 5.0 |
| 02:01~07:02~07:02~08:01 | 0.00161 | 4.9 |
| 03:01~07:02~07:02~13:02 | 0.00160 | 4.9 |
| 02:05~06:02~50:01~03:01 | 0.00158 | 4.9 |
| 02:01~15:02~51:01~13:02 | 0.00158 | 4.8 |
| 01:01~04:01~44:03~07:01 | 0.00156 | 4.8 |
| 01:01~04:01~35:01~15:01 | 0.00154 | 4.7 |
| 03:01~12:03~18:01~16:01 | 0.00153 | 4.7 |
| 24:02~07:01~08:01~03:01 | 0.00152 | 4.7 |
| 01:01~07:01~08:01~08:01 | 0.00151 | 4.6 |
| 11:01~15:02~51:01~07:01 | 0.00147 | 4.5 |
| 02:01~12:03~39:01~12:01 | 0.00143 | 4.4 |
| 24:02~04:01~35:03~16:01 | 0.00142 | 4.4 |
| 03:01~07:02~07:02~13:01 | 0.00140 | 4.3 |
| 11:01~03:03~55:01~15:01 | 0.00137 | 4.2 |
| 32:01~02:02~27:05~15:01 | 0.00136 | 4.2 |
| 02:01~14:02~51:01~07:01 | 0.00134 | 4.1 |
| 03:01~07:01~18:01~01:01 | 0.00133 | 4.1 |
| 03:01~01:02~51:01~04:02 | 0.00133 | 4.1 |
| 01:01~06:02~57:01~16:01 | 0.00132 | 4.0 |
| 02:01~03:04~40:01~13:01 | 0.00131 | 4.0 |
| 24:02~07:01~18:01~11:01 | 0.00131 | 4.0 |
| 03:01~12:03~38:01~13:01 | 0.00131 | 4.0 |
| 02:01~15:02~51:01~10:01 | 0.00131 | 4.0 |
| 03:01~05:01~44:02~04:03 | 0.00131 | 4.0 |
| 01:01~07:02~08:01~03:01 | 0.00131 | 4.0 |
| 31:01~04:01~35:08~03:01 | 0.00131 | 4.0 |
| 68:01~07:04~51:01~13:02 | 0.00131 | 4.0 |
| 02:01~05:01~44:02~12:01 | 0.00131 | 4.0 |
| 02:01~12:03~38:01~10:01 | 0.00131 | 4.0 |
| 02:01~07:02~07:02~14:01:01g | 0.00131 | 4.0 |
| 26:01~08:03~48:01~12:01 | 0.00131 | 4.0 |
| 02:01~02:02~51:01~07:01 | 0.00131 | 4.0 |
| 11:01~03:04~40:01~04:05 | 0.00131 | 4.0 |
| 33:03~03:02~58:01~03:01 | 0.00131 | 4.0 |
| 26:01~12:03~07:02~15:01 | 0.00131 | 4.0 |
| 11:01~04:01~35:01~01:03 | 0.00131 | 4.0 |
| 24:02~04:01~35:01~13:01 | 0.00131 | 4.0 |
| 29:02~16:01~44:03~07:01 | 0.00131 | 4.0 |
| 01:01~07:01~58:01~04:04 | 0.00131 | 4.0 |
| 23:01~06:02~50:01~11:02 | 0.00131 | 4.0 |
| 02:01~12:03~39:04~12:01 | 0.00131 | 4.0 |
| 02:01~16:02~51:01~16:01 | 0.00131 | 4.0 |
| 02:01~01:02~51:01~04:01 | 0.00131 | 4.0 |
| 03:01~01:02~56:01~15:01 | 0.00130 | 4.0 |
| 24:02~12:03~39:01~16:01 | 0.00130 | 4.0 |
| 23:01~06:02~50:01~03:01 | 0.00130 | 4.0 |

| **Istria & Primorje (N=865)** |  |  |
| --- | --- | --- |
| **Haplotypes HLA-A*~C*~B*~DRB1* sorted by frequency** | **frequency** | **copies** |
| 01:01~07:01~08:01~03:01 | 0.05296 | 91.6 |
| 03:01~07:02~07:02~15:01 | 0.01464 | 25.3 |
| 02:01~07:01~18:01~11:04 | 0.01412 | 24.4 |
| 11:01~04:01~35:01~01:01 | 0.01199 | 20.7 |
| 03:01~04:01~35:01~01:01 | 0.01153 | 19.9 |
| 24:02~06:02~13:02~07:01 | 0.00942 | 16.3 |
| 02:01~06:02~13:02~07:01 | 0.00881 | 15.2 |
| 02:01~07:01~08:01~03:01 | 0.00869 | 15.0 |
| 23:01~04:01~44:03~07:01 | 0.00809 | 14.0 |
| 26:01~12:03~38:01~04:02 | 0.00800 | 13.8 |
| 02:01~06:02~57:01~07:01 | 0.00786 | 13.6 |
| 11:01~07:01~18:01~11:04 | 0.00760 | 13.1 |
| 02:01~02:02~27:05~01:01 | 0.00731 | 12.6 |
| 24:02~07:02~07:02~15:01 | 0.00708 | 12.2 |
| 25:01~12:03~18:01~15:01 | 0.00693 | 12.0 |
| 02:01~07:02~07:02~15:01 | 0.00652 | 11.3 |
| 02:05~06:02~50:01~07:01 | 0.00636 | 11.0 |
| 24:02~04:01~35:02~11:04 | 0.00634 | 11.0 |
| 01:01~06:02~57:01~07:01 | 0.00580 | 10.0 |
| 02:01~02:02~27:02~16:01 | 0.00576 | 10.0 |
| 01:01~07:01~08:01~15:01 | 0.00573 | 9.9 |
| 02:01~03:03~15:01~13:01 | 0.00564 | 9.8 |
| 02:01~04:01~35:03~11:04 | 0.00525 | 9.1 |
| 01:01~12:02~52:01~15:02 | 0.00520 | 9.0 |
| 03:01~07:02~07:02~11:01 | 0.00514 | 8.9 |
| 33:01~08:02~14:02~01:02 | 0.00514 | 8.9 |
| 03:01~04:01~35:01~11:04 | 0.00462 | 8.0 |
| 68:01~12:03~39:01~16:01 | 0.00459 | 7.9 |
| 02:01~04:01~35:01~01:01 | 0.00451 | 7.8 |
| 24:02~07:01~08:01~03:01 | 0.00450 | 7.8 |
| 26:01~12:03~38:01~13:01 | 0.00446 | 7.7 |
| 02:01~05:01~44:02~04:01 | 0.00405 | 7.0 |
| 02:01~12:03~18:01~16:01 | 0.00403 | 7.0 |
| 02:01~04:01~35:03~16:01 | 0.00402 | 6.9 |
| 02:01~03:04~15:01~04:01 | 0.00400 | 6.9 |
| 01:01~07:02~07:02~15:01 | 0.00397 | 6.9 |
| 03:01~07:01~18:01~11:04 | 0.00371 | 6.4 |
| 02:01~02:02~51:01~11:01 | 0.00358 | 6.2 |
| 02:01~12:03~38:01~13:01 | 0.00350 | 6.1 |
| 03:01~12:03~18:01~16:01 | 0.00349 | 6.0 |
| 01:01~07:01~49:01~13:02 | 0.00347 | 6.0 |
| 02:01~15:02~51:01~01:01 | 0.00347 | 6.0 |
| 25:01~12:03~18:01~03:01 | 0.00347 | 6.0 |
| 32:01~04:01~35:01~16:01 | 0.00347 | 6.0 |
| 02:01~17:03~41:02~13:03 | 0.00347 | 6.0 |
| 02:01~03:02~58:01~13:02 | 0.00346 | 6.0 |
| 02:01~07:01~18:01~01:01 | 0.00345 | 6.0 |
| 02:01~15:02~51:01~11:01 | 0.00339 | 5.9 |
| 02:01~12:02~52:01~15:02 | 0.00320 | 5.5 |
| 68:01~04:01~35:03~11:12 | 0.00289 | 5.0 |
| 01:01~07:01~08:01~08:01 | 0.00289 | 5.0 |
| 32:01~02:02~40:02~16:02 | 0.00289 | 5.0 |
| 02:01~02:02~44:05~16:01 | 0.00289 | 5.0 |
| 02:01~12:03~38:01~15:01 | 0.00289 | 5.0 |
| 11:01~15:02~51:01~04:04 | 0.00288 | 5.0 |
| 02:01~12:03~39:01~16:01 | 0.00283 | 4.9 |
| 02:01~01:02~51:01~11:01 | 0.00269 | 4.6 |
| 02:01~01:02~27:05~01:01 | 0.00260 | 4.5 |
| 02:01~07:01~18:01~11:01 | 0.00252 | 4.4 |
| 02:01~15:02~51:01~07:01 | 0.00251 | 4.3 |
| 02:01~04:01~35:03~08:01 | 0.00241 | 4.2 |
| 33:01~08:02~14:02~03:01 | 0.00238 | 4.1 |
| 03:01~07:02~07:02~03:01 | 0.00236 | 4.1 |
| 02:01~02:02~27:05~11:01 | 0.00234 | 4.0 |
| 24:02~15:02~51:01~11:01 | 0.00231 | 4.0 |
| 02:01~05:01~44:02~13:01 | 0.00231 | 4.0 |
| 01:01~06:02~35:02~11:04 | 0.00231 | 4.0 |
| 01:01~08:02~14:02~07:01 | 0.00231 | 4.0 |
| 03:01~07:04~44:27~16:01 | 0.00231 | 4.0 |
| 32:01~01:02~51:01~13:01 | 0.00231 | 4.0 |
| 02:01~01:02~51:01~08:01 | 0.00231 | 4.0 |
| 24:02~15:02~51:01~07:01 | 0.00231 | 4.0 |
| 30:04~08:02~14:01~04:04 | 0.00231 | 4.0 |
| 02:01~15:02~51:01~13:01 | 0.00231 | 4.0 |
| 02:01~04:01~15:01~13:02 | 0.00231 | 4.0 |
| 02:01~14:02~51:01~11:01 | 0.00231 | 4.0 |
| 01:01~06:02~37:01~10:01 | 0.00230 | 4.0 |
| 24:02~07:02~07:02~01:01 | 0.00229 | 4.0 |

| **Central Croatia (N=1021)** |  |  |
| --- | --- | --- |
| **Haplotypes HLA-A*~C*~B*~DRB1* sorted by frequency** | **frequency** | **copies** |
| 01:01~07:01~08:01~03:01 | 0.04284 | 87.5 |
| 02:01~07:01~18:01~11:04 | 0.02084 | 42.6 |
| 03:01~07:02~07:02~15:01 | 0.01782 | 36.4 |
| 02:01~06:02~13:02~07:01 | 0.01463 | 29.9 |
| 02:01~02:02~27:02~16:01 | 0.01426 | 29.1 |
| 23:01~04:01~44:03~07:01 | 0.01035 | 21.1 |
| 26:01~12:03~38:01~04:02 | 0.01015 | 20.7 |
| 03:01~04:01~35:01~01:01 | 0.01011 | 20.6 |
| 02:01~07:02~07:02~15:01 | 0.00987 | 20.2 |
| 25:01~12:03~18:01~15:01 | 0.00642 | 13.1 |
| 02:01~12:03~38:01~13:01 | 0.00633 | 12.9 |
| 11:01~04:01~35:01~01:01 | 0.00608 | 12.4 |
| 33:01~08:02~14:02~01:02 | 0.00588 | 12.0 |
| 03:01~07:01~18:01~11:04 | 0.00583 | 11.9 |
| 24:02~04:01~35:02~11:04 | 0.00539 | 11.0 |
| 02:01~07:04~44:27~16:01 | 0.00535 | 10.9 |
| 02:01~01:02~27:05~01:01 | 0.00509 | 10.4 |
| 02:01~02:02~27:05~01:01 | 0.00502 | 10.2 |
| 02:01~02:02~40:02~11:01 | 0.00496 | 10.1 |
| 02:01~01:02~51:01~04:02 | 0.00481 | 9.8 |
| 02:01~06:02~57:01~07:01 | 0.00454 | 9.3 |
| 02:01~03:04~40:01~13:02 | 0.00452 | 9.2 |
| 01:01~12:02~52:01~15:02 | 0.00441 | 9.0 |
| 26:01~12:03~38:01~13:02 | 0.00434 | 8.9 |
| 11:01~04:01~35:01~14:01:01g | 0.00429 | 8.8 |
| 01:01~06:02~57:01~07:01 | 0.00420 | 8.6 |
| 02:05~06:02~50:01~07:01 | 0.00413 | 8.4 |
| 02:01~02:02~27:05~07:01 | 0.00401 | 8.2 |
| 03:01~04:01~35:03~01:01 | 0.00385 | 7.9 |
| 24:02~07:02~07:02~15:01 | 0.00382 | 7.8 |
| 03:01~07:02~07:02~11:01 | 0.00377 | 7.7 |
| 01:01~07:01~18:01~11:04 | 0.00370 | 7.5 |
| 02:01~12:03~18:01~16:01 | 0.00351 | 7.2 |
| 02:01~06:02~50:01~07:01 | 0.00346 | 7.1 |
| 33:01~08:02~14:02~03:01 | 0.00343 | 7.0 |
| 24:02~12:03~38:01~13:01 | 0.00343 | 7.0 |
| 02:01~03:03~15:01~11:01 | 0.00342 | 7.0 |
| 02:01~12:03~35:03~14:01:01g | 0.00340 | 6.9 |
| 29:02~16:01~44:03~07:01 | 0.00334 | 6.8 |
| 02:01~14:02~51:01~11:01 | 0.00332 | 6.8 |
| 24:02~06:02~13:02~07:01 | 0.00323 | 6.6 |
| 03:01~07:02~07:02~04:01 | 0.00305 | 6.2 |
| 02:01~02:02~27:05~11:01 | 0.00301 | 6.1 |
| 02:01~02:02~51:01~04:04 | 0.00294 | 6.0 |
| 32:01~02:02~40:02~11:01 | 0.00294 | 6.0 |
| 26:01~12:03~39:01~16:01 | 0.00294 | 6.0 |
| 24:02~03:03~15:01~04:02 | 0.00294 | 6.0 |
| 24:02~07:01~08:01~03:01 | 0.00291 | 5.9 |
| 32:01~07:01~08:01~03:01 | 0.00291 | 5.9 |
| 24:02~15:02~51:01~11:01 | 0.00282 | 5.8 |
| 03:01~02:02~27:02~16:01 | 0.00281 | 5.7 |
| 24:02~07:01~18:01~01:01 | 0.00280 | 5.7 |
| 32:01~02:02~40:02~13:01 | 0.00274 | 5.6 |
| 02:01~04:01~35:03~11:01 | 0.00269 | 5.5 |
| 30:01~06:02~13:02~07:01 | 0.00267 | 5.4 |
| 02:01~03:04~15:01~04:01 | 0.00261 | 5.3 |
| 03:01~07:02~07:02~14:01:01g | 0.00257 | 5.3 |
| 24:02~07:02~07:02~11:01 | 0.00251 | 5.1 |
| 03:01~07:04~44:27~16:01 | 0.00247 | 5.1 |
| 02:01~04:01~44:03~07:01 | 0.00246 | 5.0 |
| 02:01~12:03~39:01~16:01 | 0.00245 | 5.0 |
| 02:01~12:03~38:01~07:01 | 0.00245 | 5.0 |
| 33:01~03:02~58:01~13:02 | 0.00245 | 5.0 |
| 02:01~12:03~39:04~12:01 | 0.00245 | 5.0 |
| 23:01~06:02~50:01~03:01 | 0.00245 | 5.0 |
| 02:01~01:02~51:01~13:01 | 0.00245 | 5.0 |
| 02:01~05:01~44:02~16:01 | 0.00245 | 5.0 |
| 25:01~12:03~18:01~04:01 | 0.00245 | 5.0 |
| 02:01~05:01~44:02~04:01 | 0.00245 | 5.0 |
| 68:01~07:01~18:01~16:01 | 0.00245 | 5.0 |
| 03:01~15:02~51:01~13:02 | 0.00240 | 4.9 |
| 02:01~02:02~27:05~15:01 | 0.00235 | 4.8 |
| 02:01~16:01~44:03~07:01 | 0.00231 | 4.7 |
| 26:01~12:03~38:01~13:01 | 0.00216 | 4.4 |
| 02:01~12:03~38:01~11:01 | 0.00206 | 4.2 |
| 02:01~15:02~51:01~07:01 | 0.00204 | 4.2 |
| 01:01~15:02~51:01~11:01 | 0.00203 | 4.1 |
| 03:01~12:03~51:01~11:04 | 0.00202 | 4.1 |
| 01:01~07:02~07:02~15:01 | 0.00199 | 4.1 |
| 11:01~03:03~55:01~16:01 | 0.00196 | 4.0 |
| 02:01~14:02~51:01~07:01 | 0.00196 | 4.0 |
| 68:01~07:04~44:27~11:01 | 0.00196 | 4.0 |
| 68:01~15:02~51:01~13:01 | 0.00196 | 4.0 |
| 11:01~15:02~51:01~04:04 | 0.00196 | 4.0 |
| 24:02~02:02~44:05~01:01 | 0.00196 | 4.0 |
| 02:01~17:03~41:02~13:03 | 0.00196 | 4.0 |
| 11:01~12:02~52:01~01:01 | 0.00196 | 4.0 |
| 33:01~08:02~14:02~15:01 | 0.00196 | 4.0 |
| 32:01~12:03~38:01~15:01 | 0.00196 | 4.0 |
| 24:02~02:02~27:05~01:01 | 0.00196 | 4.0 |
| 02:01~02:02~27:05~04:04 | 0.00196 | 4.0 |
| 68:01~12:03~38:01~15:01 | 0.00196 | 4.0 |
| 11:01~12:03~35:03~04:08 | 0.00196 | 4.0 |
| 02:01~15:02~51:01~08:01 | 0.00196 | 4.0 |
| 02:01~06:02~57:01~16:01 | 0.00196 | 4.0 |
| 02:01~05:01~44:02~13:01 | 0.00196 | 4.0 |
| 26:01~06:02~57:01~11:01 | 0.00196 | 4.0 |
| 02:01~03:03~35:01~08:01 | 0.00196 | 4.0 |
| 01:01~07:01~58:01~08:04 | 0.00196 | 4.0 |
| 24:02~02:02~51:01~11:04 | 0.00196 | 4.0 |
| 02:01~14:02~51:01~01:01 | 0.00196 | 4.0 |
| 01:01~06:02~57:01~14:01:01g | 0.00196 | 4.0 |
| 66:01~17:03~41:02~13:03 | 0.00195 | 4.0 |
| 24:02~04:01~35:01~13:01 | 0.00194 | 4.0 |

| **North Croatia (N=1049)** |  |  |
| --- | --- | --- |
| **Haplotypes HLA-A*~C*~B*~DRB1* sorted by frequency** | **frequency** | **copies** |
| 01:01~07:01~08:01~03:01 | 0.04560 | 95.7 |
| 02:01~07:04~44:27~16:01 | 0.02528 | 53.0 |
| 02:01~02:02~27:02~16:01 | 0.02285 | 47.9 |
| 03:01~07:02~07:02~15:01 | 0.01438 | 30.2 |
| 02:01~07:01~18:01~11:04 | 0.01421 | 29.8 |
| 03:01~04:01~35:01~01:01 | 0.01419 | 29.8 |
| 02:01~03:04~15:01~04:01 | 0.01032 | 21.6 |
| 02:01~06:02~13:02~07:01 | 0.00948 | 19.9 |
| 02:01~02:02~27:05~01:01 | 0.00868 | 18.2 |
| 33:01~08:02~14:02~01:02 | 0.00810 | 17.0 |
| 11:01~04:01~35:01~01:01 | 0.00800 | 16.8 |
| 02:01~07:02~07:02~15:01 | 0.00794 | 16.7 |
| 23:01~04:01~44:03~07:01 | 0.00742 | 15.6 |
| 11:01~04:01~35:01~16:01 | 0.00712 | 14.9 |
| 24:02~04:01~35:02~11:04 | 0.00705 | 14.8 |
| 33:01~08:02~14:02~03:01 | 0.00682 | 14.3 |
| 24:02~07:02~07:02~15:01 | 0.00638 | 13.4 |
| 02:01~07:01~08:01~03:01 | 0.00632 | 13.3 |
| 02:01~14:02~51:01~16:01 | 0.00625 | 13.1 |
| 25:01~12:03~18:01~15:01 | 0.00572 | 12.0 |
| 30:01~06:02~13:02~07:01 | 0.00523 | 11.0 |
| 02:01~07:02~07:02~07:01 | 0.00477 | 10.0 |
| 01:01~06:02~57:01~07:01 | 0.00429 | 9.0 |
| 03:01~07:02~07:02~01:01 | 0.00421 | 8.8 |
| 02:01~15:02~51:01~11:01 | 0.00403 | 8.5 |
| 02:01~07:01~18:01~14:01:01g | 0.00402 | 8.4 |
| 02:01~04:01~35:01~01:01 | 0.00398 | 8.4 |
| 02:01~04:01~35:08~16:01 | 0.00381 | 8.0 |
| 01:01~12:02~52:01~15:02 | 0.00381 | 8.0 |
| 03:01~02:02~27:02~16:01 | 0.00353 | 7.4 |
| 03:01~04:01~35:01~11:04 | 0.00353 | 7.4 |
| 02:01~04:01~35:01~14:01:01g | 0.00348 | 7.3 |
| 02:01~05:01~44:02~15:01 | 0.00344 | 7.2 |
| 26:01~12:03~38:01~03:01 | 0.00340 | 7.1 |
| 11:01~12:02~52:01~01:01 | 0.00330 | 6.9 |
| 24:02~07:02~07:02~16:01 | 0.00326 | 6.8 |
| 03:01~07:01~08:01~03:01 | 0.00325 | 6.8 |
| 68:01~07:01~18:01~16:01 | 0.00322 | 6.7 |
| 02:01~07:01~18:01~11:01 | 0.00313 | 6.6 |
| 02:01~04:01~35:03~11:01 | 0.00312 | 6.5 |
| 11:01~07:01~18:01~11:04 | 0.00311 | 6.5 |
| 02:01~04:01~35:01~11:04 | 0.00305 | 6.4 |
| 02:01~05:01~44:02~04:01 | 0.00300 | 6.3 |
| 32:01~07:01~18:01~11:04 | 0.00295 | 6.2 |
| 03:01~07:02~07:02~16:01 | 0.00292 | 6.1 |
| 24:02~06:02~13:02~07:01 | 0.00289 | 6.1 |
| 32:01~03:03~15:01~13:01 | 0.00286 | 6.0 |
| 68:01~07:01~49:01~13:02 | 0.00286 | 6.0 |
| 02:01~03:04~40:01~13:02 | 0.00286 | 6.0 |
| 01:01~04:01~35:02~11:04 | 0.00284 | 5.9 |
| 24:02~03:03~15:01~13:01 | 0.00282 | 5.9 |
| 02:01~03:03~15:01~04:01 | 0.00275 | 5.8 |
| 02:01~04:01~35:01~16:01 | 0.00271 | 5.7 |
| 02:01~05:01~44:02~03:01 | 0.00269 | 5.7 |
| 02:01~12:03~38:01~11:04 | 0.00258 | 5.4 |
| 24:02~02:02~40:02~11:01 | 0.00252 | 5.3 |
| 24:02~12:03~18:01~11:04 | 0.00247 | 5.2 |
| 03:01~07:02~07:02~13:01 | 0.00245 | 5.1 |
| 02:01~07:02~07:02~11:01 | 0.00242 | 5.1 |
| 01:01~07:01~08:01~13:01 | 0.00242 | 5.1 |
| 02:01~02:02~27:05~11:01 | 0.00238 | 5.0 |
| 02:01~03:04~40:01~04:04 | 0.00238 | 5.0 |
| 24:02~06:02~57:01~07:01 | 0.00238 | 5.0 |
| 66:01~17:03~41:02~13:03 | 0.00238 | 5.0 |
| 01:01~07:01~15:17~13:02 | 0.00238 | 5.0 |
| 11:01~15:02~51:01~04:04 | 0.00238 | 5.0 |
| 02:01~15:02~51:01~13:01 | 0.00238 | 5.0 |
| 02:01~04:01~15:01~01:01 | 0.00238 | 5.0 |
| 02:01~12:03~18:01~01:01 | 0.00231 | 4.8 |
| 11:01~04:01~35:01~14:01:01g | 0.00229 | 4.8 |
| 02:01~01:02~27:05~01:01 | 0.00225 | 4.7 |
| 26:01~12:03~38:01~13:01 | 0.00222 | 4.7 |
| 24:02~04:01~35:01~08:01 | 0.00220 | 4.6 |
| 02:01~04:01~15:01~07:01 | 0.00214 | 4.5 |
| 03:01~07:02~07:02~11:04 | 0.00208 | 4.4 |
| 01:01~07:01~08:01~11:04 | 0.00208 | 4.4 |
| 24:02~07:02~07:02~01:01 | 0.00201 | 4.2 |
| 02:01~02:02~40:02~11:01 | 0.00200 | 4.2 |
| 02:01~15:02~51:01~14:01:01g | 0.00200 | 4.2 |
| 02:01~03:04~40:01~16:01 | 0.00198 | 4.2 |
| 25:01~12:03~18:01~04:01 | 0.00192 | 4.0 |
| 01:01~07:01~08:01~08:01 | 0.00191 | 4.0 |
| 32:01~04:01~35:01~16:01 | 0.00191 | 4.0 |
| 32:01~17:01~41:01~03:01 | 0.00191 | 4.0 |
| 01:01~06:02~37:01~10:01 | 0.00191 | 4.0 |
| 24:02~02:02~44:05~11:01 | 0.00191 | 4.0 |
| 02:01~17:03~41:02~13:03 | 0.00191 | 4.0 |
| 26:01~14:02~51:01~16:01 | 0.00191 | 4.0 |
| 32:01~02:02~40:02~11:01 | 0.00191 | 4.0 |
| 66:01~17:03~41:02~01:01 | 0.00191 | 4.0 |
| 01:01~03:03~15:01~13:01 | 0.00191 | 4.0 |
| 24:02~06:02~13:02~13:02 | 0.00191 | 4.0 |
| 02:01~07:04~44:27~14:01:01g | 0.00191 | 4.0 |
| 02:05~06:02~50:01~07:01 | 0.00191 | 4.0 |
| 02:01~12:03~38:01~11:01 | 0.00191 | 4.0 |
| 25:01~12:03~18:01~16:01 | 0.00191 | 4.0 |
| 11:01~12:03~35:03~04:08 | 0.00191 | 4.0 |
| 24:02~07:02~39:06~08:01 | 0.00191 | 4.0 |
| 30:01~06:02~13:02~13:01 | 0.00191 | 4.0 |
| 26:01~03:03~55:01~15:01 | 0.00191 | 4.0 |
| 01:01~07:01~49:01~07:01 | 0.00190 | 4.0 |
| 25:01~07:01~08:01~03:01 | 0.00190 | 4.0 |

| **East Croatia (N=1877)** |  |  |
| --- | --- | --- |
| **Haplotypes HLA-A*~C*~B*~DRB1* sorted by frequency** | **frequency** | **copies** |
| 01:01~07:01~08:01~03:01 | 0.05853 | 219.7 |
| 02:01~07:01~18:01~11:04 | 0.01724 | 64.7 |
| 03:01~07:02~07:02~15:01 | 0.01428 | 53.6 |
| 02:01~06:02~13:02~07:01 | 0.01069 | 40.1 |
| 23:01~04:01~44:03~07:01 | 0.01046 | 39.3 |
| 02:01~02:02~27:05~01:01 | 0.00991 | 37.2 |
| 02:01~02:02~27:02~16:01 | 0.00936 | 35.1 |
| 25:01~12:03~18:01~15:01 | 0.00855 | 32.1 |
| 26:01~12:03~38:01~13:01 | 0.00730 | 27.4 |
| 02:01~07:04~44:27~16:01 | 0.00711 | 26.7 |
| 03:01~04:01~35:01~01:01 | 0.00678 | 25.5 |
| 26:01~12:03~38:01~04:02 | 0.00574 | 21.6 |
| 02:01~07:02~07:02~15:01 | 0.00569 | 21.4 |
| 11:01~07:01~18:01~11:04 | 0.00568 | 21.3 |
| 11:01~04:01~35:01~01:01 | 0.00561 | 21.1 |
| 33:01~08:02~14:02~01:02 | 0.00559 | 21.0 |
| 24:02~06:02~13:02~07:01 | 0.00521 | 19.6 |
| 02:01~06:02~57:01~16:01 | 0.00499 | 18.7 |
| 30:01~06:02~13:02~07:01 | 0.00454 | 17.1 |
| 01:01~12:02~52:01~15:02 | 0.00421 | 15.8 |
| 01:01~06:02~57:01~07:01 | 0.00408 | 15.3 |
| 11:01~04:01~35:01~16:01 | 0.00406 | 15.3 |
| 01:01~07:01~18:01~11:04 | 0.00404 | 15.2 |
| 02:01~05:01~44:02~04:01 | 0.00401 | 15.1 |
| 02:01~03:04~15:01~04:01 | 0.00400 | 15.0 |
| 02:01~04:01~35:01~01:01 | 0.00393 | 14.8 |
| 29:02~16:01~44:03~07:01 | 0.00373 | 14.0 |
| 11:01~15:02~51:01~04:04 | 0.00364 | 13.7 |
| 02:01~01:02~51:01~11:01 | 0.00337 | 12.7 |
| 02:01~14:02~51:01~08:01 | 0.00334 | 12.6 |
| 02:01~07:01~08:01~03:01 | 0.00330 | 12.4 |
| 02:01~05:01~44:02~13:01 | 0.00321 | 12.1 |
| 02:01~12:03~18:01~16:01 | 0.00320 | 12.0 |
| 01:01~06:02~13:02~07:01 | 0.00319 | 12.0 |
| 02:01~03:03~15:01~13:01 | 0.00315 | 11.8 |
| 02:01~12:02~52:01~15:02 | 0.00315 | 11.8 |
| 03:01~07:02~07:02~01:01 | 0.00312 | 11.7 |
| 68:01~04:01~35:03~08:01 | 0.00293 | 11.0 |
| 02:05~06:02~50:01~07:01 | 0.00293 | 11.0 |
| 02:01~01:02~51:01~04:02 | 0.00292 | 11.0 |
| 68:01~12:03~39:01~16:01 | 0.00287 | 10.8 |
| 03:01~07:01~18:01~11:04 | 0.00275 | 10.3 |
| 02:01~15:02~51:01~11:01 | 0.00270 | 10.1 |
| 26:01~07:02~07:02~15:01 | 0.00265 | 10.0 |
| 02:01~02:02~51:01~11:01 | 0.00264 | 9.9 |
| 02:01~12:03~38:01~13:01 | 0.00262 | 9.8 |
| 02:01~06:02~57:01~07:01 | 0.00254 | 9.5 |
| 02:01~05:01~44:02~11:04 | 0.00250 | 9.4 |
| 02:01~04:01~35:03~11:01 | 0.00242 | 9.1 |
| 11:01~12:03~07:02~15:01 | 0.00240 | 9.0 |
| 66:01~17:03~41:02~13:03 | 0.00240 | 9.0 |
| 24:02~05:01~44:02~16:01 | 0.00238 | 8.9 |
| 24:02~07:02~07:02~15:01 | 0.00236 | 8.9 |
| 02:01~03:04~40:01~13:02 | 0.00235 | 8.8 |
| 32:01~12:03~39:01~16:01 | 0.00234 | 8.8 |
| 02:01~07:02~07:02~08:01 | 0.00234 | 8.8 |
| 02:01~02:02~44:05~16:01 | 0.00234 | 8.8 |
| 26:01~01:02~27:05~01:01 | 0.00233 | 8.7 |
| 02:01~03:03~15:01~04:01 | 0.00232 | 8.7 |
| 24:02~04:01~35:01~01:01 | 0.00228 | 8.6 |
| 24:02~07:01~18:01~11:01 | 0.00227 | 8.5 |
| 24:02~07:01~18:01~11:04 | 0.00219 | 8.2 |
| 01:01~02:02~27:05~01:01 | 0.00216 | 8.1 |
| 02:01~12:03~38:01~15:01 | 0.00214 | 8.0 |
| 02:01~05:01~44:02~15:01 | 0.00214 | 8.0 |
| 11:01~02:02~27:05~01:01 | 0.00213 | 8.0 |
| 24:02~04:01~35:01~11:04 | 0.00212 | 8.0 |
| 33:01~08:02~14:02~03:01 | 0.00209 | 7.8 |
| 24:02~03:03~15:01~13:01 | 0.00206 | 7.7 |
| 03:01~07:01~18:01~01:01 | 0.00202 | 7.6 |
| 03:01~04:01~35:03~13:01 | 0.00199 | 7.5 |
| 24:02~15:02~51:01~11:01 | 0.00198 | 7.4 |
| 02:01~02:02~40:02~11:01 | 0.00195 | 7.3 |
| 01:01~06:02~37:01~15:01 | 0.00190 | 7.1 |
| 24:02~04:01~35:02~11:04 | 0.00189 | 7.1 |
| 68:01~02:02~27:02~16:01 | 0.00187 | 7.0 |
| 02:01~12:03~35:03~14:01:01g | 0.00186 | 7.0 |
| 11:01~03:03~55:01~15:01 | 0.00186 | 7.0 |
| 68:02~04:01~53:01~13:02 | 0.00186 | 7.0 |
| 24:02~03:04~40:01~15:01 | 0.00184 | 6.9 |
| 03:01~07:02~07:02~13:02 | 0.00175 | 6.6 |
| 24:02~07:02~07:02~11:01 | 0.00174 | 6.5 |
| 24:02~07:01~08:01~03:01 | 0.00170 | 6.4 |
| 01:01~04:01~35:02~11:04 | 0.00167 | 6.3 |
| 02:01~15:02~51:01~13:01 | 0.00166 | 6.2 |
| 24:02~02:02~40:02~11:01 | 0.00165 | 6.2 |
| 03:01~04:01~35:03~15:01 | 0.00164 | 6.2 |
| 11:01~04:01~35:01~14:01:01g | 0.00163 | 6.1 |
| 02:01~07:02~07:02~14:01:01g | 0.00160 | 6.0 |
| 68:01~07:01~18:01~16:01 | 0.00160 | 6.0 |
| 02:01~02:02~44:05~01:01 | 0.00160 | 6.0 |
| 68:01~04:01~35:01~01:01 | 0.00160 | 6.0 |
| 01:01~07:02~08:01~03:01 | 0.00160 | 6.0 |
| 24:02~04:01~35:01~07:01 | 0.00160 | 6.0 |
| 03:02~04:01~35:03~13:02 | 0.00160 | 6.0 |
| 02:01~15:02~51:01~07:01 | 0.00160 | 6.0 |
| 02:01~06:02~44:02~11:01 | 0.00160 | 6.0 |
| 68:02~08:02~14:02~13:03 | 0.00160 | 6.0 |
| 24:02~01:02~51:01~04:03 | 0.00160 | 6.0 |
| 02:01~02:02~27:05~04:04 | 0.00160 | 6.0 |
| 02:01~12:03~18:01~01:01 | 0.00157 | 5.9 |
| 02:01~03:03~15:01~11:03 | 0.00156 | 5.9 |
| 02:01~01:02~27:05~01:01 | 0.00156 | 5.9 |
| 11:01~04:01~35:01~13:01 | 0.00156 | 5.9 |
| 02:01~12:03~38:01~16:01 | 0.00154 | 5.8 |
| 02:01~12:03~51:01~11:04 | 0.00151 | 5.7 |
| 02:01~01:02~51:01~14:01:01g | 0.00151 | 5.7 |
| 11:01~04:01~35:01~11:01 | 0.00150 | 5.6 |
| 02:01~07:01~18:01~04:01 | 0.00150 | 5.6 |
| 01:01~04:01~35:03~11:01 | 0.00149 | 5.6 |
| 24:02~02:02~44:05~01:01 | 0.00149 | 5.6 |
| 02:01~04:01~35:01~14:01:01g | 0.00148 | 5.6 |
| 25:01~12:03~18:01~10:01 | 0.00147 | 5.5 |
| 11:01~12:02~52:01~15:02 | 0.00142 | 5.3 |
| 11:01~06:02~57:01~07:01 | 0.00142 | 5.3 |
| 68:01~07:01~08:01~03:01 | 0.00140 | 5.2 |
| 01:01~12:03~38:01~16:01 | 0.00139 | 5.2 |
| 11:01~12:03~39:01~16:01 | 0.00139 | 5.2 |
| 02:01~04:01~15:01~01:01 | 0.00137 | 5.2 |
| 02:01~04:01~35:03~16:01 | 0.00137 | 5.1 |
| 03:01~07:01~08:01~03:01 | 0.00136 | 5.1 |
| 02:01~14:02~51:01~01:01 | 0.00135 | 5.1 |
| 02:01~15:02~51:01~01:01 | 0.00135 | 5.1 |
| 11:01~01:02~56:01~01:01 | 0.00135 | 5.1 |
| 02:01~07:02~07:02~01:01 | 0.00135 | 5.1 |
| 02:01~12:03~39:06~16:01 | 0.00133 | 5.0 |
| 02:01~07:01~18:01~11:01 | 0.00133 | 5.0 |
| 32:01~12:03~51:01~01:01 | 0.00133 | 5.0 |
| 33:01~08:02~14:02~01:01 | 0.00133 | 5.0 |
| 03:01~04:01~35:01~04:01 | 0.00133 | 5.0 |
| 24:03~07:02~07:02~15:02 | 0.00133 | 5.0 |
| 30:04~08:02~14:01~04:04 | 0.00133 | 5.0 |
| 02:01~06:02~57:01~15:01 | 0.00133 | 5.0 |
| 02:05~07:01~49:01~07:01 | 0.00133 | 5.0 |
| 11:01~04:01~35:01~13:03 | 0.00133 | 5.0 |
| 01:01~07:01~15:17~13:02 | 0.00133 | 5.0 |
| 24:02~06:02~13:02~10:01 | 0.00133 | 5.0 |
| 32:01~02:02~40:02~16:02 | 0.00133 | 5.0 |
| 24:02~14:02~51:01~13:01 | 0.00132 | 5.0 |
| 02:01~05:01~44:02~14:01:01g | 0.00131 | 4.9 |
| 02:01~12:03~38:01~04:02 | 0.00131 | 4.9 |
| 01:01~07:01~49:01~14:01:01g | 0.00130 | 4.9 |
| 26:01~12:03~38:01~14:01:01g | 0.00130 | 4.9 |
| 24:02~12:03~35:03~01:01 | 0.00130 | 4.9 |
| 24:02~06:02~50:01~07:01 | 0.00129 | 4.8 |
| 01:01~02:02~27:05~03:01 | 0.00128 | 4.8 |
| 02:01~04:01~44:03~07:01 | 0.00126 | 4.7 |
| 02:01~02:02~51:01~13:01 | 0.00126 | 4.7 |
| 03:01~12:03~18:01~16:01 | 0.00124 | 4.7 |
| 03:01~01:02~51:01~11:01 | 0.00124 | 4.6 |
| 02:01~12:03~39:01~11:01 | 0.00124 | 4.6 |
| 02:01~12:03~38:01~07:01 | 0.00123 | 4.6 |
| 01:01~07:01~08:01~01:01 | 0.00123 | 4.6 |
| 02:01~17:03~41:02~13:03 | 0.00123 | 4.6 |
| 02:01~07:01~18:01~15:01 | 0.00120 | 4.5 |
| 31:01~07:02~07:02~15:01 | 0.00119 | 4.5 |
| 25:01~07:01~18:01~11:04 | 0.00119 | 4.5 |
| 02:01~14:02~51:01~11:01 | 0.00116 | 4.4 |
| 24:02~04:01~35:03~11:01 | 0.00116 | 4.3 |
| 24:02~07:02~07:02~13:02 | 0.00115 | 4.3 |
| 03:01~07:02~07:02~13:01 | 0.00115 | 4.3 |
| 02:01~12:03~39:01~16:01 | 0.00114 | 4.3 |
| 01:01~07:02~07:02~15:01 | 0.00113 | 4.3 |
| 03:01~04:01~35:03~03:01 | 0.00112 | 4.2 |
| 32:01~02:02~40:02~13:01 | 0.00110 | 4.1 |
| 03:01~07:02~07:02~11:01 | 0.00109 | 4.1 |
| 24:02~04:01~44:03~07:01 | 0.00109 | 4.1 |
| 25:01~12:03~18:01~04:01 | 0.00109 | 4.1 |
| 03:01~12:03~38:01~07:01 | 0.00107 | 4.0 |
| 01:01~06:02~37:01~07:01 | 0.00107 | 4.0 |
| 02:01~03:04~40:01~11:04 | 0.00107 | 4.0 |
| 24:02~02:02~27:05~01:01 | 0.00107 | 4.0 |
| 01:01~02:02~40:02~16:02 | 0.00107 | 4.0 |
| 03:01~03:03~55:01~14:01:01g | 0.00107 | 4.0 |
| 24:02~03:03~55:01~16:01 | 0.00107 | 4.0 |
| 26:01~12:03~07:02~15:01 | 0.00107 | 4.0 |
| 02:01~03:04~40:01~04:04 | 0.00107 | 4.0 |
| 03:01~12:03~35:03~14:01:01g | 0.00107 | 4.0 |
| 01:01~03:03~15:01~11:01 | 0.00107 | 4.0 |
| 26:01~04:01~35:03~07:01 | 0.00107 | 4.0 |
| 02:01~15:02~51:01~13:02 | 0.00107 | 4.0 |
| 23:01~06:02~50:01~03:01 | 0.00107 | 4.0 |
| 26:01~07:01~49:01~01:01 | 0.00107 | 4.0 |
| 03:01~08:02~14:02~01:02 | 0.00107 | 4.0 |
| 32:01~04:01~35:01~14:01:01g | 0.00107 | 4.0 |
| 68:01~15:02~51:01~15:01 | 0.00107 | 4.0 |
| 11:01~12:02~52:01~01:01 | 0.00107 | 4.0 |
| 03:01~06:02~47:01~07:01 | 0.00107 | 4.0 |
| 02:01~02:02~27:02~13:05 | 0.00107 | 4.0 |
| 24:02~12:03~51:01~04:03 | 0.00107 | 4.0 |
| 68:01~07:04~44:27~11:01 | 0.00106 | 4.0 |
| 02:01~07:01~49:01~13:02 | 0.00106 | 4.0 |
| 01:01~07:01~08:01~07:01 | 0.00106 | 4.0 |
| 01:01~06:02~37:01~11:01 | 0.00106 | 4.0 |
| 24:02~07:01~18:01~14:01:01g | 0.00106 | 4.0 |
